# Inhibition of 5-Lipoxygenase in Hepatic Stellate Cells Alleviates Liver Fibrosis

**DOI:** 10.3389/fphar.2021.628583

**Published:** 2021-02-18

**Authors:** Shiyun Pu, Yanping Li, Qinhui Liu, Xu Zhang, Lei Chen, Rui Li, Jinhang Zhang, Tong Wu, Qin Tang, Xuping Yang, Zijing Zhang, Ya Huang, Jiangying Kuang, Hong Li, Min Zou, Wei Jiang, Jinhan He

**Affiliations:** ^1^ Department of Pharmacy and State Key Laboratory of Biotherapy, West China Hospital, Sichuan University, Chengdu, China; ^2^ Laboratory of Clinical Pharmacy and Adverse Drug Reaction, West China Hospital, Sichuan University, Chengdu, China; ^3^ Tianjin Key Laboratory of Metabolic Diseases and Department of Physiology, Tianjin Medical University, Tianjin, China; ^4^ Molecular Medicine Research Center, West China Hospital of Sichuan University, Chengdu, China

**Keywords:** liver fibrosis, non-alcoholic steatohepatitis, α-SMA, ERK, zileuton

## Abstract

**Background and Purpose:** Activation of hepatic stellate cells (HSC) is a central driver of liver fibrosis. 5-lipoxygenase (5-LO) is the key enzyme that catalyzes arachidonic acid into leukotrienes. In this study, we examined the role of 5-LO in HSC activation and liver fibrosis.

**Main Methods:** Culture medium was collected from quiescent and activated HSC for target metabolomics analysis. Exogenous leukotrienes were added to culture medium to explore their effect in activating HSC. Genetic ablation of 5-LO in mice was used to study its role in liver fibrosis induced by CCl_4_ and a methionine-choline-deficient (MCD) diet. Pharmacological inhibition of 5-LO in HSC was used to explore the effect of this enzyme in HSC activation and liver fibrosis.

**Key Results:** The secretion of LTB_4_ and LTC_4_ was increased in activated vs. quiescent HSC. LTB_4_ and LTC_4_ contributed to HSC activation by activating the extracellular signal-regulated protein kinase pathway. The expression of 5-LO was increased in activated HSC and fibrotic livers of mice. Ablation of 5-LO in primary HSC inhibited both mRNA and protein expression of fibrotic genes. *In vivo*, ablation of 5-LO markedly ameliorated the CCl_4_- and MCD diet-induced liver fibrosis and liver injury. Pharmacological inhibition of 5-LO in HSC by targeted delivery of the 5-LO inhibitor zileuton suppressed HSC activation and improved CCl_4_- and MCD diet-induced hepatic fibrosis and liver injury. Finally, we found increased 5-LO expression in patients with non-alcoholic steatohepatitis and liver fibrosis.

**Conclusion:** 5-LO may play a critical role in activating HSC; genetic ablation or pharmacological inhibition of 5-LO improved CCl_4_-and MCD diet-induced liver fibrosis.

## Introduction

Liver fibrosis is a common outcome of chronic liver injury such as chronic hepatotoxicity and non-alcoholic steatohepatitis (NASH) (Yu et al., 2018). If unmanaged, liver fibrosis can advance to cirrhosis and portal hypertension and often requires liver transplantation.

Hepatic stellate cells (HSC) play a key role in the formation of hepatic fibrosis (Higashi et al., 2017). In normal liver, HSC stay quiescent (Ogawa et al., 2007). With injurious stimuli, HSC transdifferentiate from a quiescent to activated state (Puche et al., 2013), becoming proliferative and producing a high amount of α-smooth muscle actin (α-SMA) and collagens (De Minicis et al., 2008). When this extracellular matrix accumulates excessively, it causes a fibrotic outcome and scars on the liver (Wu et al., 2018). The mechanism of HSC activation is not fully understood. Several signaling pathways participate in activating HSC (Woodhoo et al., 2012). Transforming growth factor-β (TGF-β) induces the phosphorylation of Smad2/3, which in turn promotes HSC activation and regulates the expression of fibrotic genes (Feng and Derynck, 2005). Activation of extracellular signal-regulated kinase (ERK) also contributes to HSC activation (Chen et al., 2016; Xie et al., 2017).

Arachidonic acid is the precursor of biologically and clinically important eicosanoids (Harizi et al., 2008). 5-lipoxygenase (5-LO) is the key enzyme that catalyzes arachidonic acid into leukotrienes (Alexander et al., 2011). Upon activation of 5-LO-activating protein (Flap), 5-LO oxidases arachidonic acid to the unstable intermediate 5-hydroperoxyeicosatetraenoic acid (5-HPETE), which is further dehydrated to form leukotriene A4 (LTA_4_) (Silverman and Drazen, 1999). LTA_4_ is converted to LTB_4_ via LTA_4_ hydrolase enzymes or to LTC_4_ via LTC_4_ synthase (Hofmann and Steinhilber, 2013). Both LTB_4_ and LTC_4_ are inflammatory lipid mediators that have important effects on the development of allergic rhinitis, bronchial asthma and atherosclerosis (Kowal et al., 2017). Inhibiting 5-LO by an inhibitor such as zileuton or blocking the effect of leukotrienes by their receptor antagonist such as montelukast, have been clinically used for asthma treatment (De Corso et al., 2019).

Recent studies indicated that the 5-LO pathway is associated with fibrosis (Qian et al., 2015; Su et al., 2016). 5-LO is expressed in human dermal fibroblasts, synovial fibroblasts, pulmonary fibroblasts and rat adventitial fibroblasts (Lin et al., 2014; Su et al., 2016). Activation of these cells can be restrained by 5-LO inhibitors (Lin et al., 2014; Su et al., 2016). LTB_4_ and LTC_4_ are secreted from lung fibroblasts (Shiratori et al., 1989; Paiva et al., 2010) and contribute to the proliferation and migration of these cells (Hirata et al., 2013). However, the role of 5-LO in HSC activation and liver fibrosis remains unknown.

In this study, we first used metabolomics to reveal that LTB_4_ and LTC_4_ are highly secreted during the activation of HSC. Secreted LTB_4_ and LTC_4_ promoted HSC activation via an ERK signaling pathway. Ablation or inhibition of 5-LO could suppress HSC activation. In mouse fibrotic models, ablation or targeted inhibition of 5-LO in HSC relieved liver fibrosis and injury. Finally, we found increased expression of 5-LO in liver sections of patients with NASH and fibrosis.

## Materials and Methods

### CCl_4_- and Methionine-Choline–Deficient Diet-Induced Models of Liver Fibrosis

For CCl_4_-induced liver fibrosis, 8-week-old C57 BL/6J (WT) and 5-lipoxygenase knockout (5-LO^−/−^) mice received an intraperitoneal (i.p.) injection of CCl_4_ (1 ml/kg body weight) twice a week. For MCD diet-induced liver fibrosis, WT and 5-LO^−/−^ mice were fed a methionine-choline-supplied (MCS) or MCD diet (TROPHIC Animal Feed High-Tech, China) for 6 weeks. For therapeutic experiments, zileuton loaded in cRGDyK (Cyclo [Arg-Gly-Asp-_D_Tyr-Lys])-guided liposome (RGD-Lip; 10 mg/kg) or vehicle was given by tail vein injection once every 3 days during the last 4 weeks of CCl_4_ or MCD diet treatment. All mice were housed at West China Hospital, Sichuan University in accordance with the guidelines of the animal care utilization committee of the institute. Food and water were freely available to mice, except otherwise stated.

### Preparation and Characterization of RGD-Lips

Liposome (Lip) were prepared by the thin-film hydration method as described (Li et al., 2019; Zhang et al., 2020). Zileuton-loaded RGD-Lips (RGD-Lip/zileuton) were prepared by adding zileuton to the lipid organic solution before the solvent evaporation. The mean particle size and zeta potential of Lip were measured by dynamic light scattering with the Zetasizer Nano ZS90 instrument (Malvern, United Kingdom). The morphology of Lip was examined by transmission electron microscopy (H-600, Hitachi, Japan) with 2% phosphotungstic acid staining.

### Serum Alanine Aminotransferase and Aspartate Aminotransferase Measurement

Serum ALT and AST levels were detected by using commercial kits (BioSino Bio-Technology and Science).

### Hydroxyproline Assay

An amount of 50 mg liver tissue was dissolved in acid hydrolysate in glass tube and heated in boiling water bath for 20 min. Hydroxyproline was extracted according to the manufacturer's instructions and measured by using kits (Nanjing Jiancheng Bioengineering Institute).

### Histology Analysis

The left lobe of the mouse liver was removed and immediately fixed in 10% neutral-buffered formalin, embedded in paraffin, and sectioned at 4 μm. Liver sections were stained with hematoxylin and eosin (H&E). For picrosirius red staining, liver sections were incubated with 0.1% sirius red in saturated picric acid for 60 min at room temperature. The Sirus Red positive area were detected by Image J. Fibrosis was assessed by picrosirius red staining according to the Ishak fibrosis criteria (Ishak et al., 1995).

### Isolation and Culture of HSC

HSC were isolated from livers of WT mice and 5-LO^−/−^ mice via *in situ* collagenase perfusion and underwent differential centrifugation on Optiprep (Sigma) density gradients, as described (Kwon et al., 2014). Isolated HSC were cultured in collagen-coated dishes with DMEM supplemented with 10% fetal bovine serum and antibiotics at 37°C. The purity of HSC was >95% as determined by their typical star-like shape and abundant lipid droplets.

### Measurement of Zileuton Concentration in Different Types of Liver Cells

Mice were injected with CCl_4_ to cause liver fibrosis and treated with RGD-Lip/zileuton (10 mg/kg) for 4 h. Primary hepatocytes were isolated as described (Chen et al., 2019). The remaining cells were divided into three groups, fixed, perforated and stained with anti-α-SMA (HSC), anti-F4/80 (Kupffer cells) and anti-CD31 antibodies. HSC, Kupffer cells and LSECs were sorted by flow cytometry. Biliary epithelial cells were isolated as previously described (Li et al., 2019). Before extraction, cells were added to 30 μl ddH_2_O and underwent repeated freezing and thawing 5 times. A 25-μl aliquot of samples was added to a 1.5-ml polypropylene tube followed by 200 μl methanol. The mixture was vortex-mixed for 5 min and centrifuged at 14,000 rpm for 10 min at room temperature. The top layer was transferred to a new 1.5 ml polypropylene tube and evaporated to vacuum dryness at 37°C. Samples were re-dissolved with 50 μl 80% methanol. The mixture was vortex-mixed for 30 s and centrifuged at 14,000 rpm for 5 min at 4 °C. The 40-μl supernatant was transferred to a 250 μl polypropylene autosampler vial and sealed with a Teflon crimp cap. Partially purified cell samples were analyzed by using LC-MS/MS.

### Systematic Metabolomic Analysis of Arachidonic Acid in 5-Lipoxygenase Pathway

Serum-free supernatant from primary mouse HSC were collected in an ice bath and extracted by solid-phase extraction. The metabolomics of arachidonic acid were detected and analyzed as described (Zhang et al., 2015).

### LTB_4_ and LTC_4_ Measurement

Serum-free primary mouse HSC were collected in an ice bath and protected from light. Samples were centrifuged (600 × g, 5  min, 4°C), and with the resulting supernatant, LTB_4_ and LTC_4_ levels were determined by using ELISA kits (Cayman Chemical).

### Immunofluorescence Staining

Primary HSC were fixed in 4% paraformaldehyde for 15 min, incubated in 0.2% Triton X-100 1×PBS for 15 min for permeabilization of cytomembrane. Antigens in paraffin sections were repaired by microwaving in 0.01 M citrate buffer (pH = 6.0) for 15 min. Both cells and tissue samples were incubated with antibodies in 4°C for 12 h. Immunoreactive compounds were incubated in room temperature for 1 h for conjugating with fluorescence-labeling secondary antibodies. All antibodies used in these experiments are in Supplementary Supplementary Table S1.

### Western Blot Analysis

Western blot analysis was performed as described (Chen et al., 2019). The antibodies were listed in supplementary Supplementary Table S1. The expression of β-tubulin was a loading control. Immunoreactive bands were visualized on nitrocellulose membranes by using fluorescence-conjugated secondary antibodies (LI-COR, United States). The relative density was calculated by the ratio of the density of the protein of interest to β-tubulin.

### Real-Time PCR Analyses

Total RNA isolation and PT-PCR was performed as described (Chen et al., 2019). The primers for detected genes are in Supplementary Table S2.

### Statistical Analysis

Experiments were repeated at least 3 times with similar results. Quantitative results are expressed as the mean ± SEM. Statistical significance was determined by Student’s unpaired two-tailed *t* test or one-way ANOVA multiple comparison test as indicated in legends. *p* < 0.05 was considered statistically significant.

## Results

### LTB_4_ and LTC_4_ were Enriched in Supernatant of A-HSC

**FIGURE 1:**
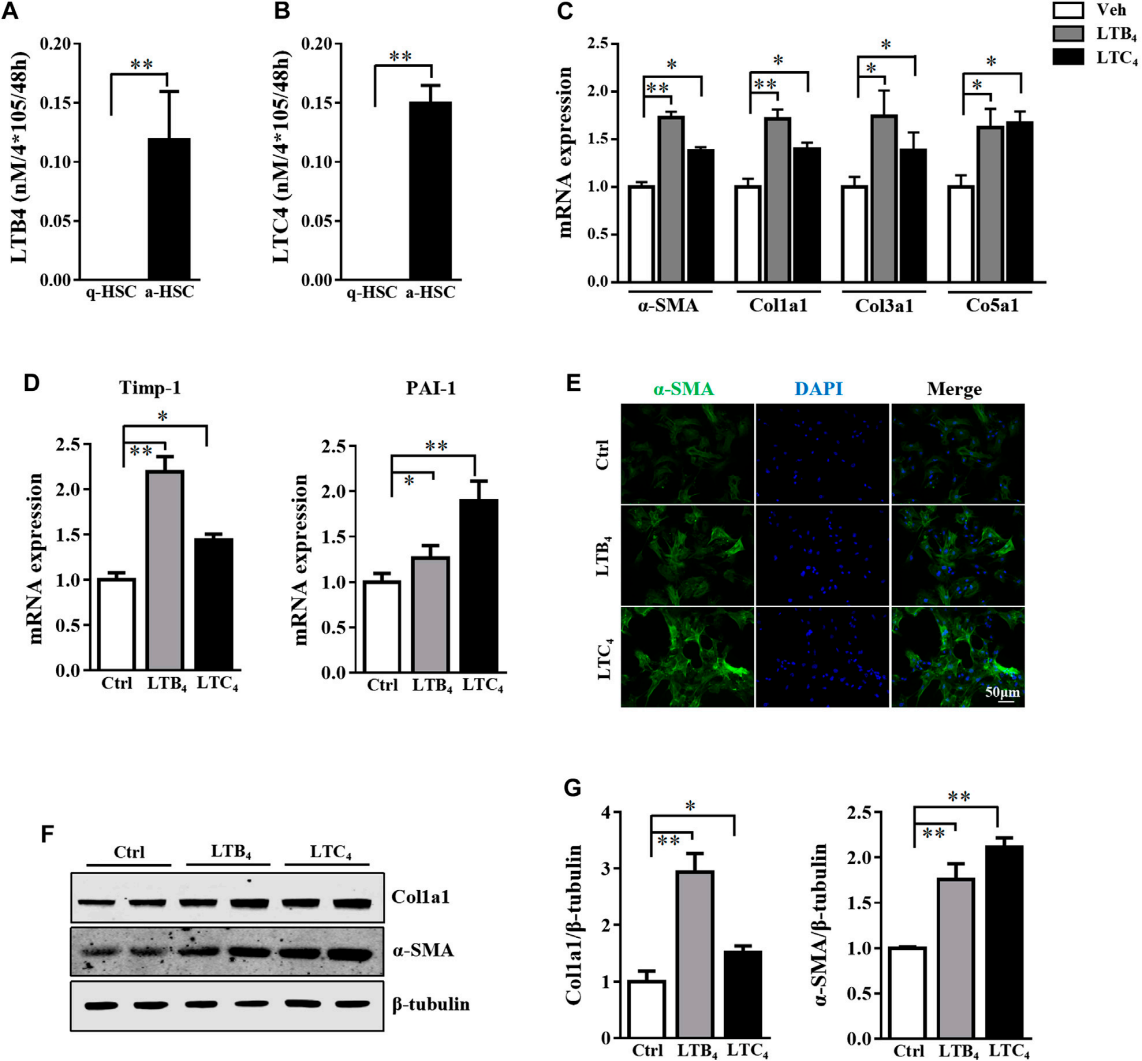
LTB_4_ and LTC_4_ levels were elevated during HSC activation and promote HSC activation. **(A,B)** Primary HSC were isolated from wild-type (WT) mice and cultured for 2 days (quiescent HSC [q-HSC]) or 7 days (activated HSC [a-HSC]). ELISA detection of secretion of LTB_4_ and LTC_4_ in supernatant from q-HSC or a-HSC. **(C–G)** Primary HSC were isolated from 5-LO^−/−^ mice and cultured in DMEM with high glucose with 10% heat-inactivated fetal bovine serum for 2 days. Cells were co-cultured with vehicle (Veh), LTB_4_, or LTC_4_ for 4 days. **(C,D)** qRT-PCR analysis of mRNA levels of fibrotic genes. **(E)** Immunofluorescence analysis of effect of LTB_4_ or LTC_4_ treatment on α-SMA expression. **(F,G)** The protein level of α-SMA and Col1a1 protein. Data are mean ± SEM, n = 5, *p < 0.05, **p < 0.01.

To explore the potential role of lipoxygenase pathway in arachidonic acid during HSC activation, we collected cell supernatants from quiescent HSC (q-HSC) and activated HSC (a-HSC, culture activated for 7 days) (Thi Thanh Hai et al., 2018). Among metabolites identified in the lipoxygenase pathway, target metabolomics revealed that LTB_4_ level was selectively increased in a-HSC (Supplementary Figure S1). ELISA further confirmed the increased LTB_4_ level in the supernatant of a-HSC (Figure 1A). Consistently, the level of LTC_4_, another metabolite of 5-LO, was also increased (Figure 1B).

### LTB_4_ and LTC_4_ Contributed to Activating HSC via Phosphorylation of the ERK Pathway

**FIGURE 2:**
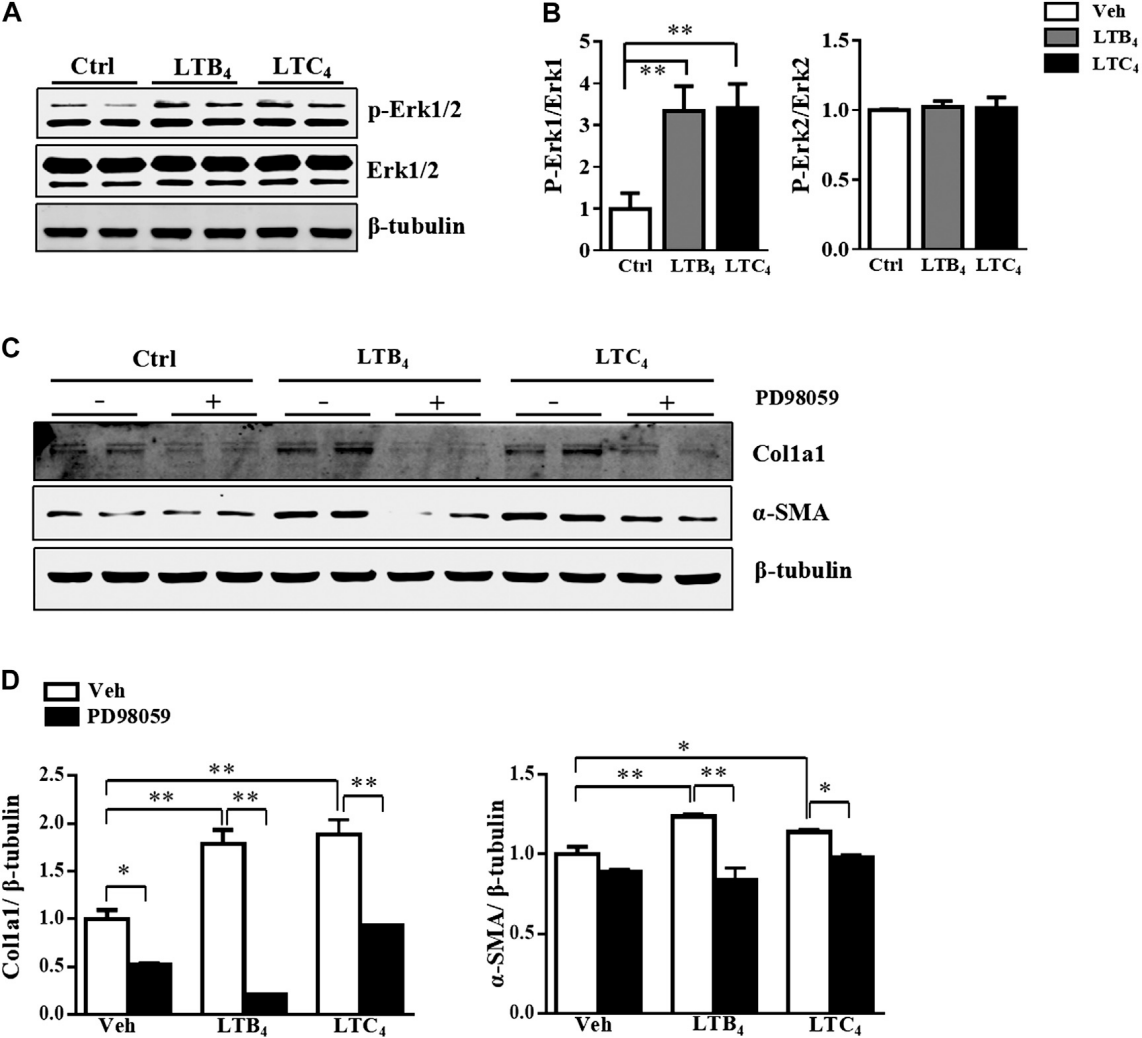
LTB_4_ and LTC_4_ promote HSC activation via ERK signaling pathway. **(A,B)** The protein levels of p-ERK, t-ERK and β-tubulin in HSC after treatment with LTB_4_, LTC_4_ or TGF-β1 for 30 min **(C,D)** Western blot analysis of protein levels of α-SMA and Col1a1 in primary HSC isolated from 5-LO^−/−^ mice and pre-treated with PD98059 before administration of LTB_4_ or LTC_4_. Data are mean ± SEM, n = 5, *p < 0.05, **p < 0.01.

The high level of LTB_4_ and LTC_4_ released by a-HSC prompted us to explore their function in activating HSC. To exclude the influence of endogenous LTB_4_ and LTC_4_, we isolated primary HSC from 5-LO^−/−^ mice and added exogenous LTB_4_ or LTC_4_ to these cells for 4 days. Treatment with LTB_4_ or LTC_4_ significantly increased the expression of fibrotic genes including α-SMA, collagen 1a1 (Col1a1), Col3a1, Col5a1, tissue inhibitor of metalloproteinase 1 (Timp-1) and plasminogen activator inhibitor 1 (PAI-1) (Figures 1C,D). On immunofluorescence staining, LTB_4_ and LTC_4_ increased α-SMA accumulation in HSC as compared with controls (Figure 1E). Western blot analysis further confirmed that LTB_4_ and LTC_4_ elevated the protein levels of α-SMA and Col1a1 (Figures 1F,G). Therefore, LTB_4_ or LTC_4_ could promote HSC activation. We next explored the signaling pathway conveying the effect of LTB_4_ or LTC_4_. LTB_4_ or LTC_4_ seemed not to induce the phosphorylation of Smad2/3, a key pathway molecule for TGF-β-induced fibrosis (Supplementary Figure S2A). Instead, we found that LTB_4_ and LTC_4_ induced phosphorylation of the ERK1 signaling pathway (Figures 2A,B). The phosphorylation of ERK is necessary for LTB_4_- and LTC_4_-induced fibrosis because PD98059, a mitogen-activated protein kinase inhibitor, abolished their effects (Figures 2C,D; Supplementary Figure S2B). Other MAP kinases, according to p-p38 and p-JNK, were no change after LTB_4_ or LTC_4_ administration (Supplementary Figure S2C). Therefore, the effect of LTB4 and LTC4 on HSC may be mediated by ERK1 signaling.

### 5-LO was Upregulated and Promoted HSC Activation

**FIGURE 3:**
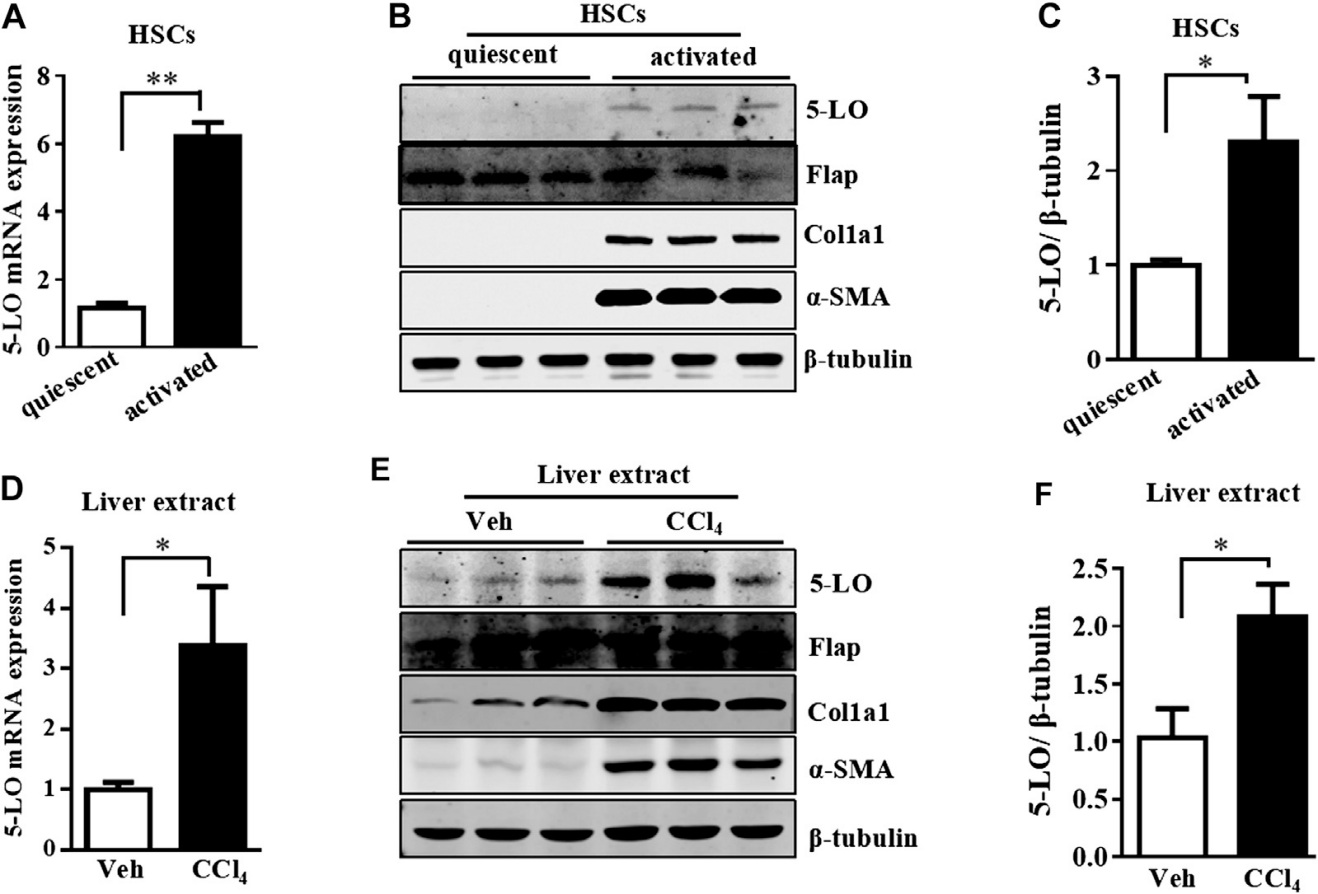
5-LO was upregulated in a-HSC and fibrotic livers. The mRNA and protein levels of 5-LO detected in q-HSC and a-HSC **(A–C)** and with CCI_4_ treatment in mouse livers **(D–F)**. The protein levels of Flap, Col1a1, α-SMA and β-tubulin were also detected in all above groups **(B,E)**.

5-LO is the key enzyme in the synthesis of LTB_4_ and LTC_4_ (Alexander et al., 2011). We further investigated whether the high level of LTB_4_ and LTC_4_ released by a-HSC resulted from the increased expression of 5-LO. The mRNA and protein levels of 5-LO were significantly upregulated in a-HSC as compared with q-HSC (Figures 3A–C). In contrast, the expression of Flap was not changed by HSC activation (Figure 3B). In CCl_4_-induced liver fibrosis, 5-LO was also significantly increased along with other fibrotic genes (Figures 3D–F). Again, Flap expression was not significantly changed (Figure 3E). Liver fibrosis occurs during the progression of non-alcoholic steatohepatitis (NASH) (Nasr et al., 2018). Both the mRNA and protein levels of 5-LO were increased in an independent model of MCD diet-induced NASH (Anstee and Goldin, 2006) (Supplementary Figures S3A–C). These results suggest that increased 5-LO level may be responsible for the high level of LTB_4_ and LTC_4_ in a-HSC.

**FIGURE 4:**
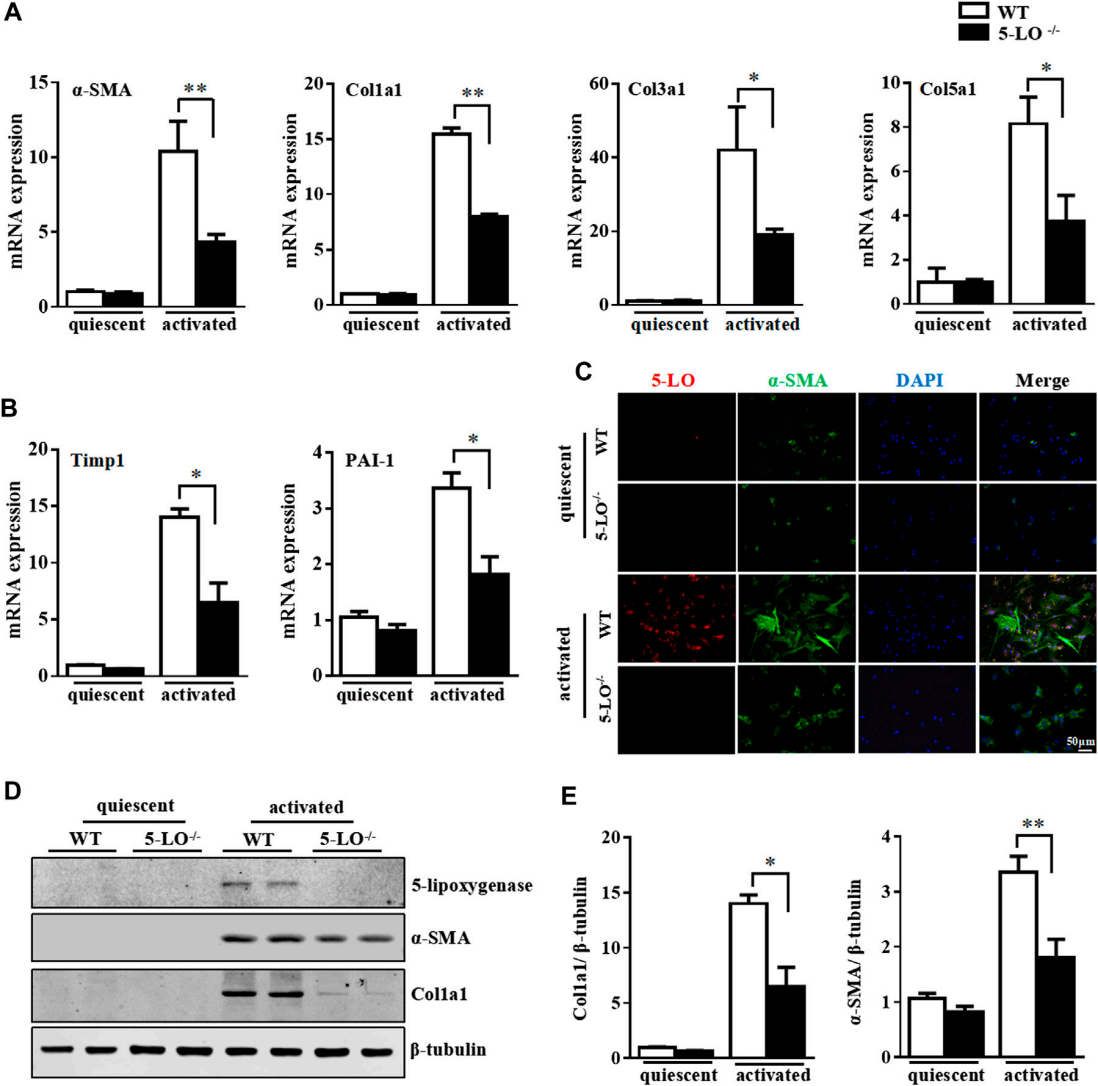
Genetic ablation of 5-LO restrained activation of primary HSC. Primary HSC were isolated from WT and 5-LO ^−/−^ mice. **(A,B)** qRT-PCR of mRNA levels of fibrosis genes. **(C)** Immunofluorescence analysis of effect of 5-LO ablation on α-SMA expression. **(D,E)** Western blot analysis of α-SMA and Col1a1 protein levels in q-HSC and a-HSC. Data are mean ± SEM, n = 5, *p < 0.05, **p < 0.01.

To further investigate the effect of 5-LO in HSC activation, we isolated primary HSC from wild-type and 5-LO^−/−^ mice, then compared the expression of fibrotic genes after culture activation. Ablation of 5-LO significantly inhibited the expression of culture-induced fibrotic genes, such as α-SMA, Col1a1, Col3a1, Col5a1, Timp1 and PAI-1 (Figures 4A,B), indicating that 5-LO was involved in HSC activation. Inhibition of HSC activation was confirmed by immunofluorescent staining, which showed the expression of α-SMA less detectable in 5-LO^−/−^ HSC (Figure 4C). Western blot analysis revealed that ablation of 5-LO suppressed α-SMA and Col1a1 expression (Figures 4D,E). The incubation of supernatant from WT a-HSC (WT-CM) was more effective to activate HSC than that from 5-LO^−/−^ a-HSC (5-LO^−/−^-CM) (Supplementary Figures S4A,B).

### Genetic Ablation of 5-LO Ameliorated CCl_4_- and MCD Diet-Induced Liver Fibrosis, Inflammation and Hepatic Injury

**FIGURE 5:**
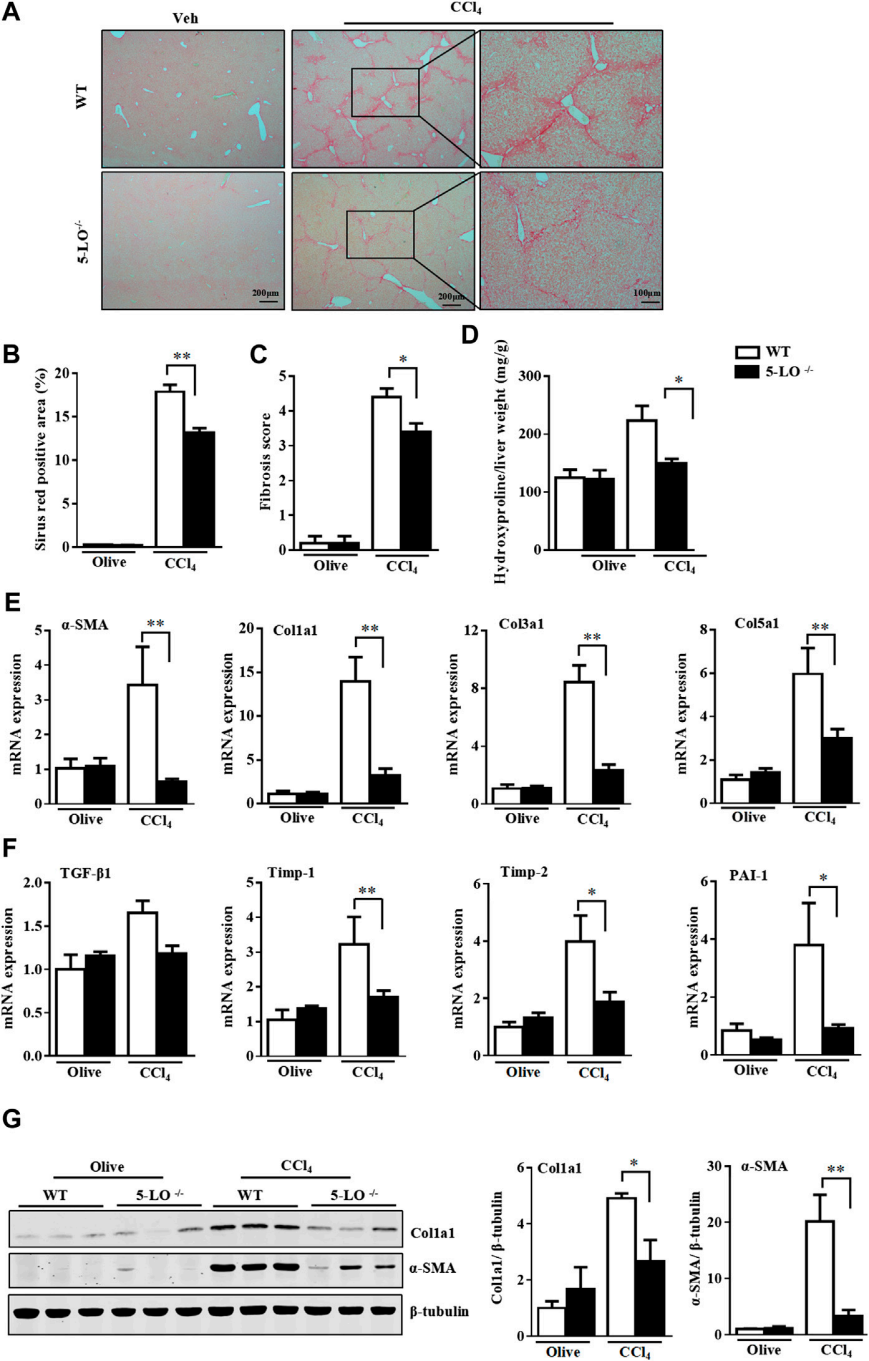
Genetic ablation of 5-LO ameliorated liver fibrosis after CCl_4_ injection. WT and 5-LO ^−/−^ mice were treated with olive oil or CCl_4_ for 8 weeks. Fibrosis stage was assessed by picrosirius red staining **(A,B)** for collagen according to the Ishak criteria **(C)**. **(D)** Detection of hepatic hydroxyproline level. **(E,F)** qRT-PCR analysis of mRNA levels of fibrosis genes in livers of 4 groups. **(G)** Western blot analysis of the protein levels of α-SMA and Col1a1. Data are mean ± SEM, n = 7, *p < 0.05, **p < 0.01.

To explore the *in vivo* function of 5-LO in liver fibrosis, we first exposed WT and 5-LO^−/−^ mice to CCl_4_ twice a week for 8 weeks by intraperitoneal injection. As expected, CCl_4_ caused significant hepatic fibrosis as compared with olive oil, as assessed by picrosirius red staining (Figures 5A,B). In contrast, 5-LO^−/−^ mice showed improved liver fibrosis and decreased hepatic fibrosis scores (Figure 5C). Consistently, the level of hydroxyproline was significantly lower in 5-LO^−/−^ than WT mice (Figure 5D), which suggests that 5-LO deletion conferred resistance to CCl_4_-induced hepatic fibrosis. Among the fibrotic markers, α-SMA, collagens, TGF-β1, Timp-1/2 and PAI-1 were greatly suppressed in 5-LO^−/−^ mice after CCl_4_ treatment (Figures 5E,F). The protein levels of α-SMA and Col1a1 were also greatly reduced in 5-LO^−/−^ mice with chronic CCl_4_ injection (Figure 5G). CCl_4_ administration showed an increasing serum levels of ALT and AST (Supplementary Figure S5A). However, this liver injury was significantly decreased in 5-LO^−/−^ mice. Chronic liver injury accelerated the accumulation of inflammatory cells around the vessels (Supplementary Figure S5B). Ablation of 5-LO greatly suppressed CCl_4_-induced inflammatory cell infiltration (Supplementary Figures S5B,C). These results were further supported by the decreased expression of inflammatory genes seen in the liver of 5-LO^−/−^ mice (Supplementary Figure S5D).

In another independent model of MCD diet-induced NASH, collagen deposition was reduced in livers of 5-LO^−/−^ mice along with decreased fibrosis scores and hydroxyproline levels (Supplementary Figures S6A–D). Improved liver fibrosis was further confirmed by gene expression and protein analysis. Indeed, 5-LO ablation decreased the mRNA levels of fibrotic genes (α-SMA, Col1a1, Col3a1, Col5a1, TGF-β1, Timp-1/2 and PAI-1) (Supplementary Figures S6E,F) and the protein levels of α-SMA and Col1a1 (Supplementary Figures S6G,H). 5-LO ablation was protective in MCD-diet induced liver injury, as indicated by reduced serum levels of ALT and AST and inflammation scores (Supplementary Figures S7A–C). Inflammation plays an important role in MCD diet-induced NASH (Locatelli et al., 2014). Consistently, the mRNA levels of inflammatory genes including tumor necrosis factor α (TNF-α), interleukin 1β (IL-1β) and monocyte chemoattractant protein 1 (Mcp-1) were decreased in 5-LO^/−^ mice (Supplementary Figure S7D). The hepatic expression of CD68, a marker of macrophages, was also reduced (Supplementary Figure S7D). Therefore, 5-LO ablation ameliorated CCl_4_- and MCD diet-induced hepatic fibrosis, inflammation and liver injury.

### Pharmacological Inhibition of 5-LO by Targeted Delivery Suppressed HSC Activation

**FIGURE 6:**
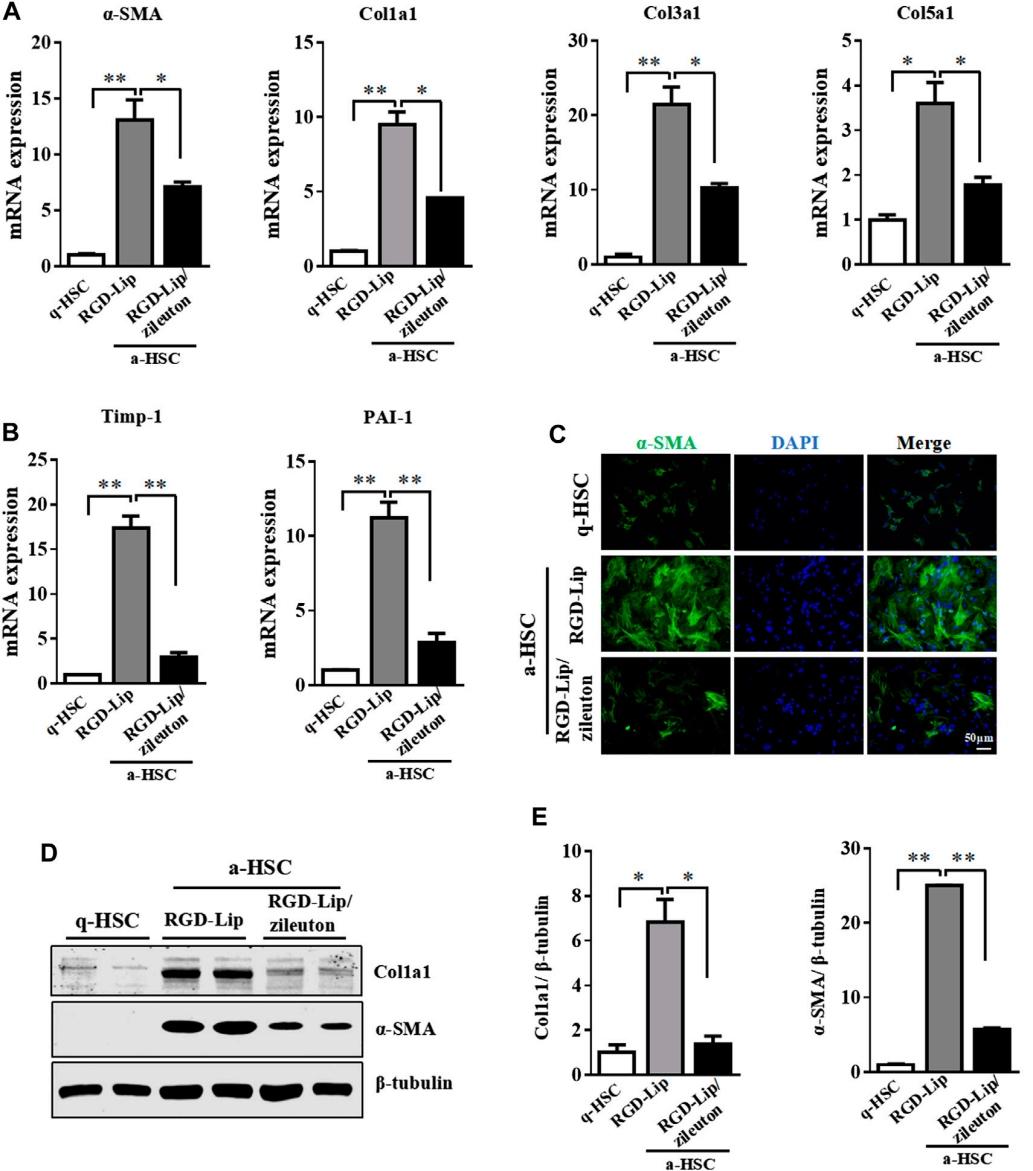
Pharmacological inhibition of 5-LO suppressed activation of HSC. Primary HSC were isolated from WT mice and cultured in DMEM with high glucose and 10% heat-inactivated fetal bovine serum for 2 days (q-HSC) or 7 days (a-HSC). Primary HSC were treated with RGD-Lip or RGD-Lip/zileuton for 48 h **(A,B)** qRT-PCR analysis of effect of RGD-Lip/zileuton treatment on the mRNA expression of fibrotic genes. **(C)** Immunofluorescence analysis of effect of RGD-Lip/zileuton treatment on α-SMA expression. **(D,E)** Western blot analysis of α-SMA and Col1a1 protein levels in q-HSC and a-HSC. Data are mean ± SEM, n = 5, *p < 0.05, **p < 0.01.

The protective effect of 5-LO ablation prompted us to explore whether pharmacological inhibition of 5-LO would have a similar effect in restraining the activation of primary HSC. We used an HSC-specific drug delivery system by modifying sterically stable liposome (Lip) with cRGDyK, a pentapeptide that binds to integrin αvβ3 on the surface of a-HSC (Li et al., 2019). cRGDyK-guided Lip showed high selectivity toward activated but not quiescent HSC, and preferentially accumulated in the fibrotic liver (Li et al., 2019; Zhang et al., 2020). We loaded with zileuton, an inhibitor of 5-LO, into this delivery system (RGD-Lip/zileuton). The schematic illustration, particle size, morphology and entrapment efficiency were comparable to regular liposome (Supplementary Figures S8A,B, Supplementary Table S3). As expected, RGD-Lip/zileuton significantly suppressed the secretion of LTB_4_ by a-HSC (Supplementary Figure S9), which indicates the inhibition of 5-LO. In a-HSC, zileuton delivered by RGD-Lip greatly inhibited the expression of fibrotic genes including α-SMA, Col1a1, Col3a1, Col5a1, Timp-1 and PAI-1 (Figures 6A,B). These effects were further confirmed by immunofluorescent staining and western blot analysis (Figures 6C–E). Therefore, pharmacological inhibition of 5-LO suppressed HSC activation.

### Targeted Delivery of Zileuton to HSC Is Efficient Against Liver Fibrosis

**FIGURE 7:**
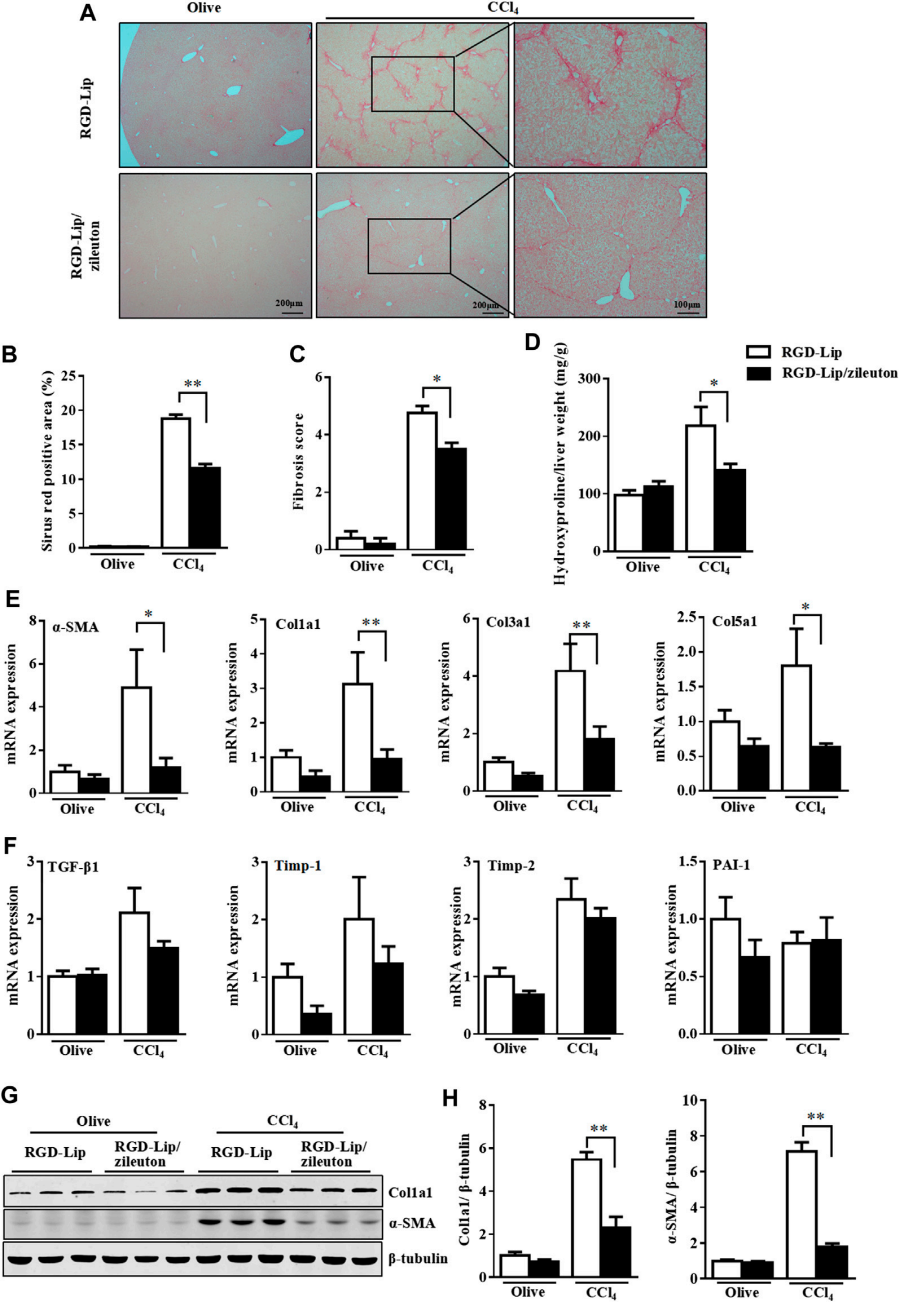
Targeted delivery of zileuton to HSC is efficient against CCl_4_-induced liver fibrosis. WT mice were treated with olive oil or CCl_4_ for 8 weeks. During the last 4 weeks, mice given RGD-Lip or RGD-Lip/zileuton (10 mg/kg) every 3 days by tail vein injection. **(A,B)** Liver sections were collected for picrosirius red staining. **(C)** Fibrosis score was assessed for collagen according to the Ishak criteria. **(D)** Detection of hepatic hydroxyproline level. **(E,F)** qRT-PCR analysis of mRNA levels of fibrotic genes in livers of 4 groups. **(G)** Western blot analysis of the protein levels of α-SMA and Col1a1. Data are mean ± SEM, n = 7, *p < 0.05, **p < 0.01.

We then evaluated the *in vivo* therapeutic effect of RGD-Lip/zileuton in CCl_4_-and MCD diet-induced liver fibrosis. In this experiment, mice were injected with CCl_4_ for 4 weeks, then treated with RGD-Lip/zileuton (10 mg/kg) every 3 days (Supplementary Figure S10A). RGD-Lip could specifically deliver zileuton to HSC because the concentration of zileuton in HSC was about 28.99-, 4.71-, 4.67- and 34.01-times higher than that in hepatocytes, Kupffer cells, endothelial cells and biliary epithelial cells, respectively (Supplementary Figure S10B). Specific inactivation of 5-LO by RGD-Lip/zileuton in HSC greatly decreased liver fibrosis as shown by picrosirius red staining and liver hydroxyproline quantification (Figures 7A–D). The expression of fibrotic genes such as α-SMA, Col1a1, Col3a1, Col5a1, Timp1 and PAI-1 was also mitigated (Figures 6E,F). Western blot analysis confirmed the reduced protein levels of Col1a1 and α-SMA (Figures 6G,H). Targeted inhibition of 5-LO in HSC also alleviated CCl_4_-induced liver injury and hepatic inflammation (Supplementary Figures S11A–C), but did not reduce the accumulation of F4/80 positive cells (Supplementary Figure S11D).

The therapeutic effects of RGD-Lip/zileuton were further demonstrated in an MCD diet-induced NASH model (Supplementary Figure S12). Specific inactivation of 5-LO in HSC explicitly alleviated MCD diet-induced liver fibrosis (Supplementary Figures S13A–D). Expression of fibrotic genes was suppressed in RGD-Lip/zileuton-treated mice (Supplementary Figures S13E,F) and α-SMA and collagen accumulation was reduced (Supplementary Figures S13G,H). Targeted delivery of zileuton also relieved liver injury in MCD-induced NASH (Supplementary Figure S14A). H&E staining and the expression of proinflammatory genes such as TNF-α and Mcp-1 indicated decreased inflammation in livers of RGD-Lip/zileuton-treated mice (Supplementary Figures S14B–D).

### Increased Expression of 5-LO in Patients with Hepatic Fibrosis

**FIGURE 8:**
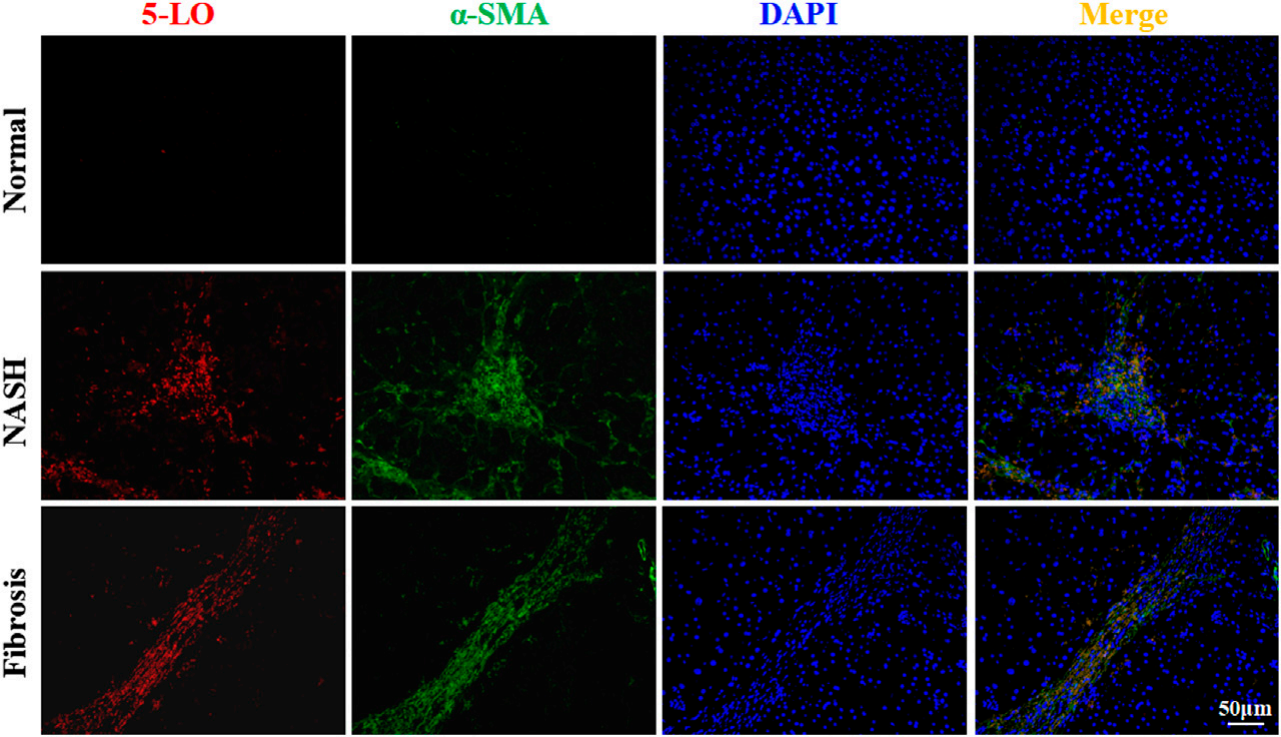
The expression of 5-LO was increased in liver sections of patients with fibrosis. Liver sections were collected from normal individuals or patients with non-alcoholic steatohepatitis or liver fibrosis and were stained with 5-LO and α-SMA. Data are mean ± SEM, n = 3.

We next determined whether 5-LO expression was changed in patients with NASH or liver fibrosis. The diagnosis of NASH and fibrosis was confirmed by H&E and picrosirius red staining (Supplementary Figures S15A–C). As compared with healthy individuals, the expression of 5-LO was increased in liver sections from patients with NASH and fibrosis (Figure 8). The expression of 5-LO was largely co-localized in α-SMA-positive cells, which were HSC (Figure 8). These results were consistent with 5-LO possibly having a positive role in activation of HSC in rodents.

## Discussion

In this study, we found increased secretion of LTB_4_ and LTC_4_ in a-HSC. LTB_4_ and LTC_4_ contributed to HSC activation via ERK signaling. Elevated LTB_4_ and LTC_4_ was likely a result of increased expression of 5-LO during HSC activation. Genetic ablation of 5-LO protected mice against CCl_4_- and MCD diet-induced fibrosis and liver injury. Pharmacological inhibition of 5-LO in HSC by targeted delivery of zileuton prevented CCl_4_- and MCD diet-induced liver fibrosis. Finally, we found 5-LO level increased in liver sections of patients with liver fibrosis.

Several reports have suggested that 5-LO may play a role in fibrosis (Titos et al., 2004). 5-LO is expressed in various fibroblast cells, such as pulmonary fibroblasts, human myofibroblasts and skin fibroblasts (Nagy et al., 2011; Xiao et al., 2011) (Su et al., 2016). Titos et al. found 5-LO expressed in Kupffer cells (Titos et al., 2004). Pharmacological inhibition of 5-LO protected mice from CCl_4_-induced liver fibrosis (Horrillo et al., 2007). However, the following study could not confirm the expression of 5-LO in Kupffer cells (Takeda et al., 2017). Also, 5-LO expression was detected in HSC isolated from mice and rats (Paiva et al., 2010; Shajari et al., 2015). However, the function of 5-LO in HSC was not known. In our study, we found 5-LO expressed in HSC, and its expression was increased during their activation. With increased expression of 5-LO, the secretion of LTB_4_ and LTC_4_ was significantly elevated in a-HSC (Figures 1A,B; Supplementary Figure S1). LTB_4_ and LTC_4_ promoted HSC activation via ERK1 signaling pathway. This finding was consistent with a previous report of LTB_4_ and LTC_4_ leading to pulmonary fibrosis because of stimulating the activation and differentiation of fibroblasts (Hirata et al., 2013). Other lipoxygenases, such as 12-lipoxygenase, was also upregulated in a-HSC compared with q-HSC (Mori et al., 2020). However, we did not found the difference of 12-HETE, a metabolic of arachidonic acid through 12-lipoxygease, in q-HSC and a-HSC supernatant (Supplementary Figure S1). The role of 12-lipoxygenase in HSC deserves further study.

Liver fibrosis homeostasis is maintained by the balance of extracellular matrix synthetic machinery, contributing to increased rate of collagen synthesis and activities of the cellular fibrinolytic system. Timp-1 belongs to the Timp family and participates in degrading the extracellular matrix (Iredale, 2007). PAI-1 is a member of the serine protease inhibitor family, the main physiological inhibitor of serine protease, and contributes to the fibrinolytic system (Wang et al., 2007). Several studies have shown that Timp-1 and PAI-1 are key factors modulating fibrolysis and extracellular matrix deposition (Iredale, 2007; Wang et al., 2007). Knockout or pharmacological inhibition of Timp1 and PAI-1 inhibited fibrosis in liver (Parsons et al., 2004). Our *in vitro* results showed significantly increased expression of Timp-1 and PAI-1 in culture-activated HSC as compared with q-HSC. However, genetic ablation of 5-LO in HSC decreased levels of Timp1 and PAI-1, which may contribute to suppressed extracellular matrix deposition. *In vivo*, 5-LO ablation or pharmacological inhibition reduced the Timp-1 and PAI-1 expression, which helped reduce hydroxyproline level in mouse liver.

Oral treatment or injection of the 5-LO inhibitor zileuton causes systemic pharmacological side effects. Zileuton could increase oxidative stress in hepatocytes and may cause hepatocyte damage (Altumina, 1995). In addition, zileuton treatment was found to increase serum ALT and AST levels (Watkins et al., 2007). These results suggest that systemic zileuton administration may cause drug-induced side effects. cRGD is pentapeptide that binds with high affinity to integrin αV and β3 which are highly expressed in a-HSC (Li et al., 2019). It was also confirmed that cRGD-guided Lips specifically target activated HSC *in vitro* and *vivo* (Li et al., 2019; Zhang et al., 2020). In agreement with a previous report, we found that RGD-Lip-delivered zileuton was highly enriched in HSC but not hepatocytes, Kupffer cells, endothelial cells or biliary cells (Supplementary Figure S9B). RGD-Lip/zileuton administration significantly protected mice against CCl_4_-and MCD diet-induced liver fibrosis (Figure 7; Supplementary Figure S12). Therefore, targeted delivery of zileuton to inhibit 5-LO by RGD-Lip may be a promising way to manage liver fibrosis.

Obviously, both CCl_4_ and MCD-diet treatments induce inflammation, which were reduced by 5-LO ablation or RGD/Lip-zileuton administration. Horrillo et al. also found that 5-LO inhibitor protected mice from CCl_4_-induced liver inflammation (Horrillo et al., 2007). In our study, knockout of 5-LO did reduce the accumulation of F4/80 positive cells in fibrotic liver (Supplementary Figure S5C). However, treatment of RGD/Lip-zileuton did not reduce the accumulation of F4/80 positive cells (Supplementary Figure S11D). It was reported that activation of HSC mediated immune response (Chou et al., 2011; Chang et al., 2013; Bigorgne et al., 2016). We speculate that the beneficial effect of RGD/Lip-zileuton is more due to reduce in the HSC fibrosis compartment than reduced inflammation in the Kupffer cell compartment. The changes of these inflammatory indicators are related to the decrease of HSC activation.

LTB_4_ and LTC_4_ were reported as lipid mediators for attracting neutrophils and for lipid accumulation (Lund et al., 2017). Induction of LTB_4_ and LTC_4_ biosynthesis might cause hepatotoxicity via neutrophil activation (Shiratori et al., 1989; Takeda et al., 2017). 5-LO is the key enzyme that catalyzes arachidonic acid to form LTB_4_ and LTC_4_ (Alexander et al., 2011). In our study, both deletion of 5-LO and targeted inhibition of 5-LO in HSC protected mice against CCl_4_-and MCD diet-induced liver injury, at least in part by reducing LTB_4_ and LTC_4_ production in the liver.

In summary, we demonstrate that 5-LO inhibition confers resistance to CCl_4_- and MCD diet-induced hepatic fibrosis. The protective effect of 5-LO deletion was partially due to decreased level of LTB_4_ as well as LTC_4_ and reduced activation of HSC. Our data show that 5-LO is critical for liver fibrosis in the setting of supporting HSC activation. 5-LO expression was also increased in HSC in liver sections of patients with fibrosis. Strategies to target inhibition of 5-LO in HSC may be useful for treating liver fibrosis.

## Data Availability

The original contributions presented in the study are included in the article/Supplementary Material. Further inquiries can be directed to the corresponding author.

## Abbreviations

ALT, alanine aminotransferase; AST, aspartate aminotransferase; 5-LO, 5-lipoxygenase; 5-HPETE, 5-hydroperoxyeicosatetraenoic acid; NASH, non-alcoholic steatohepatitis; MCD diet, methionine-choline-deficient diet; MCS diet, methionine-choline-supplied diet; CCl_4_, carbon tetrachloride; IL, interleukin; HSC, hepatic stellate cell; α-SMA, alpha smooth muscle actin; LTA4, leukotriene A4; LTB4, leukotriene B4; LTC4, leukotriene C_4_; TIMP-1/2, tissue inhibitor of metalloproteinase 1/2; TGF-β1, transforming growth factor-β1; PAI-1, plasminogen activator inhibitor 1; Flaps, 5-LO-activating proteins; ERK, extracellular signal-regulated protein kinase; MCP-1, monocyte chemoattractant protein 1; TNF-α, tumor necrosis factor alpha.
