# Supplementary material for: Inhibition of 5-Lipoxygenase in Hepatic Stellate Cells Alleviates Liver Fibrosis
Source: Front Pharmacol. 2021 Feb 18;12:628583. doi: 10.3389/fphar.2021.628583 (PMC7930623; doi:10.3389/fphar.2021.628583)

Inhibition of 5-lipoxygenase in hepatic stellate cells alleviates liver fibrosis

Shiyun Pu[1, 2#], Yanping Li[2#], Qinhui Liu[2], Xu Zhang[3], Lei Chen[1, 2], Rui Li[1, 2], Jinhang Zhang[1, 2], Tong Wu[1, 2], Qin Tang[1, 2], Xuping Yang[1, 2], Zijing Zhang[1, 2], Ya Huang[1, 2], Jiangying Kuang[1, 2], Hong Li[1, 2], Min Zou[1], Wei Jiang[4], and Jinhan He[1, 2 *]

[1]Department of Pharmacy and State Key Laboratory of Biotherapy, West China Hospital, Sichuan University. [2]Laboratory of Clinical Pharmacy and Adverse Drug Reaction, West China Hospital, Sichuan University. [3]Tianjin Key Laboratory of Metabolic Diseases and Department of Physiology, Tianjin Medical University, Tianjin, China. [4]Molecular Medicine Research Center, West China Hospital of Sichuan University, Chengdu, China, 610041.
#, these authors contributed equally to this paper.

***，Corresponding author:**
Jinhan He, Department of Pharmacy, State Key Laboratory of Biotherapy and Cancer Center, West China Hospital of Sichuan University and Collaborative Innovation Center of Biotherapy, Chengdu, Sichuan, China. Email: jinhanhe@scu.edu.cn.

Supplementary Methods
Histology analysis
Liver sections were stained with hematoxylin and eosin (H&E). Tissue sections were examined in a blinded fashion by a single pathologist and graded for steatosis, inflammation and hepatocyte ballooning as described (1).

References
1. Kleiner DE, Brunt EM, Van Natta M, Behling C, Contos MJ, Cummings OW, Ferrell LD, et al. Design and validation of a histological scoring system for nonalcoholic fatty liver disease. Hepatology 2005;41:1313-1321.

Supplementary Figure Legends

Supplementary Figure 1. LTB_4 levels were elevated during HSCs activation. Primary HSCs were isolated from wild-type (WT) mice and cultured for 2 days (quiescent HSCs [q-HSCs]) or 7 days (activated HSCs [a-HSCs]). Supernatants were collected from q-HSCs or a-HSCs for metabolomic analysis. Data are mean ± SEM, n = 5, *$p < 0.05$, **$p < 0.01$.

Supplementary Figure 2. LTB_4 and LTC_4 levels did affect the ERK pathway but not SMAD and AKT pathway.(A) Western blot analysis of protein levels of p-SMAD, SMAD, p-AKT, AKT, p-ERK1/2, ERK1/2, and β-tubulin in HSC after treatment with LTB_4, LTC_4 or TGF-β1 for 30 min. (B) Western blot analysis of the expression of p-ERK1/2 and ERK1/2 with LTB_4 or LTC_4 treatment. HSC were pre-treated with PD98059 for 1 hr then LTB_4 or LTC_4. (C) Western blot analysis of protein levels of p-P38, p-JNK, and β-tubulin after treatment with LTB_4, LTC_4 or TGF-β1 for 30 min.

Supplementary Figure 3. 5-LO was upregulated in NASH-mice liver.
(A) mRNA level of 5-LO in WT mice fed an MCS or MCD diet for 6 weeks. (B) Protein levels of 5-LO, Flap, Col1a1 and α-SMA in livers of WT mice fed an MCS and MCD diet for 6 weeks. (C) Western blot quantification of 5-LO expression in Supplementary Figure 3B. Data are mean ± SEM, n = 5, **$p < 0.01$.

Supplementary Figure 4. The effect of supernatant from 5-LO $^{-/-}$ and WT a-HSC activation on HSC activation.
Primary HSC were isolated from WT mice and 5-LO$^{-/-}$ mice and cultured for 7 days. The supernatants were collected from WT mice (WT-CM) and 5-LO$^{-/-}$ mice (5-LO$^{-/-}$-CM) and incubated with primary HSC from WT mice and 5-LO$^{-/-}$ mice for 7days. (A-B) The mRNA and protein levels of fibrotic markers were detected. Data are mean ± SEM, n = 5, *$p < 0.05$, **$p < 0.01$.

Supplementary Figure 5. Genetic ablation of 5-LO ameliorated liver injury and inflammation with CCl_4 treatment.
WT and 5-LO $^{-/-}$ mice were injected with olive oil or CCl_4 for 8 weeks. Serum level of ALT and AST levels (A) and inflammatory cell infiltration (B) with CCl_4 treatment. (C) Liver sections were stained with F4/80 and α-SMA.(D) qRT-PCR analysis of mRNA levels of TNF-α, IL-1β, Mcp-1, and Cd68. Data are mean ± SEM, n = 5, *$p < 0.05$, **$p < 0.01$.

Supplementary Figure 6. Genetic ablation of 5-LO ameliorated MCD diet-induced liver fibrosis. WT and 5-LO $^{-/-}$ mice were fed an MCS or MCD diet for 6 weeks. Fibrosis stage was assessed after picrosirius red staining (A-B) for collagen according to the Ishak criteria (C). (D) The detection of hepatic hydroxyproline levels. (E-F) qRT-PCR analysis of mRNA levels of fibrotic genes in livers of 4 groups. (G) Western blot analysis of protein levels of α-SMA and Col1a1. (H) Western blot quantification of α-SMA and Col1a1 expression in Supplementary Figure 5F. Data are mean ± SEM, n = 7, *$p < 0.05$, **$p<0.01$.

Supplementary Figure 7. Genetic ablation of 5-LO ameliorated liver injury and inflammation with MCD diet. WT and 5-LO $^{/-}$ mice were fed an MCS or MCD diet for 6 weeks. qRT-PCR analysis of the effect of genetic ablation of 5-LO on MCD diet-induced liver injury (A)

and inflammatory cell infiltration (B). (C) NAFLD Activity Score for degree of steatosis, lobular inflammation, and hepatocyte ballooning. (D) qRT-PCR analysis of mRNA levels of proinflammatory genes in livers of WT and 5-LO$^{-/-}$ mice. Data are mean ± SEM, n =7, *$p < 0.05$, **$p<0.01$.

Supplementary Figure 8. The characterization of liposomes. (A) Schematic illustration of RGD-Lip/zileuton. (B) The morphology of liposomes.

Supplementary Figure 9. RGD-Lip/zileuton inhibits secretion of LTB_4 in a-HSCs. Primary HSCs were isolated from WT mice and cultured for 7 days to activation. Cells were pretreated with RGD-Lip or RGD-Lip/zileuton (10 μM) for 48 hr. The supernatants were collected for LTB_4 detection. Data are mean ± SEM, n = 5, *$p < 0.05$.

Supplementary Figure 10. Targeted delivery of zileuton to HSCs in CCl_4-induced fibrotic model. (A) The regimen of RGD-Lip/zileuton treatment. CCl_4 injection lasted for 8 weeks. During the last 4 weeks, mice were administered RGD-Lip or RGD-Lip/zileuton (10 mg/kg) every 3 days by tail vein injection. (B) The concentration of zileuton in HSCs, hepatocytes (MPH), Kupffer cells (KC), liver sinusoidal endothelial cells (LSEC) and biliary duct epithelial cells (BEC). Data are mean ± SEM, n = 7, **$p < 0.01$.

Supplementary Figure 11. Targeted delivery of zileuton to HSC ameliorated liver injury and inflammation with CCl_4 administration. WT mice were treated as in Supplementary Figure 9A. (A) Serum was collected for ALT and AST detection. (B) Liver sections were isolated for H&E staining. (C) qRT-PCR analysis of mRNA levels of proinflammatory genes in liver. (D) Liver sections were stained with F4/80 and α-SMA.Data are mean ± SEM, n = 7, *$p < 0.05$, **$p < 0.01$.

Supplementary Figure 12. Targeted delivery of zileuton to HSC in MCD diet model. The regimen of RGD-Lip/zileuton treatment. WT mice were fed an MCS or MCD diet for 6 weeks. During the last 4 weeks, mice were treated with RGD-Lip or RGD-Lip/zileuton (10 mg/kg) every 3 days by tail vein injection.

Supplementary Figure 13. Targeted delivery of zileuton to HSC was efficient against MCD diet-induced liver fibrosis. WT mice were treated as in Supplementary Figure 11. Fibrosis stage was assessed after picrosirius red staining (A-B) for collagen according to the Ishak criteria (C). (D) Liver hydroxyproline was detected to reflect the degree of collagen deposition in the liver. (E-F) qRT-PCR analysis of mRNA levels of fibrotic genes in livers of 4 groups. (G-H) Western blot analysis of protein levels of α-SMA and Col1a1. Data are mean ± SEM, n = 7, *$p < 0.05$, **$p < 0.01$.

Supplementary Figure 14. Targeted delivery of zileuton to HSCs ameliorated liver injury and inflammation with MCD diet. WT mice were treated as in Supplementary Figure 11. Effect of inhibition of 5-LO on serum ALT and AST levels (A) and H&E staining (B) of MCD diet-induced liver injury. (C) Inhibition of 5-LO in HSCs decreased hepatic steatosis, lobular inflammation, ballooning and NAFLD score. (D) qRT-PCR analysis of mRNA levels of proinflammatory genes in livers. Data are mean ± SEM, n = 7, *$p < 0.05$, **$p < 0.01$.

Supplementary Figure 15. Human tissue for H&E and picrosirius red staining.
Livers were collected from normal individuals or patients with NASH and fibrosis for H&E (A) and picrosirius red staining (B). The Sirus red positive area were detected by Image J. Data are mean ± SEM, n = 3-5, $^{**}p < 0.01$.

Supplementary Tables

Supplementary Table 1: List of antibodies used for western blot analysis and immunostaining.

Antibodies	Application	Company	CAT. No.
**Anti-α-SMA**	WB, IF	SIGMA	A5228
**Anti-α-SMA**	IF	HUABIO	ER1003
**Anti-β-tubulin**	WB	Zen BioScience	200608
**Anti-Col1a1**	WB	Bosterbio	PB0981
**Anti-5-Lipoxygenase**	WB, IF	CST	3289
**Anti-5-Lipoxygenase**	IF	SANTA	sc-136195
**Anti-p-Erk1/2**	WB	SANTA	sc-7383
**Anti-t-Erk1/2**	WB	SANTA	sc-93
**Anti-p-Akt**	WB	SANTA	sc-7985
**Anti-t-Akt**	WB	Bosterbio	BM4400
**Anti-p-Smad2/3**	WB	CST	8828S
**Anti-t-Smad2/3**	WB	CST	5678S

Supplementary Table 2: List of specific primers used for RT-PCR

Genes	Sense (5'- 3')	anti-sense (5'- 3')
**mα-SMA**	CAGGGAGTAATGGTTGGAAT	TCTCAAACATAATCTGGGTCA
**mCol1a1**	CATGAGCCGAAGCTAACCC	TGTGGCAGATACAGATCAAGC
**mCol3a1**	TGCTCCTGTGCTTCCTGATG	GACCTGGTTGTCCTGGAAGG
**mCol5a1**	CCTGCCTGACTCATCTGTGC	AGGCTGTGGTGTGGCTAGGT
**mTGF-β1**	ATGGTGGACCGCAACAAC	AGCCACTCAGGCGTATCAG
**mTimp-1**	GCAACTCGGACCTGGTCATAA	CGGCCCGTGATGAGAAACT
**mTimp-2**	TCAGAGCCAAAGCAGTGAGC	GCCGTGTAGATAAACTCGATGTC
**mPai-1**	TTCAGCCCTTGCTTGCCTC	ACACTTTTACTCCGAAGTCGGT
**mAlox5**	CACGGGGACTACATCGAGTT	GTGCTGCTTGAGGATGTGAA
**mMcp1**	GCTGGAGAGCTACAAGAGGATC	GTCAACTTCACATTCAAAGGTGC
**mTnf-α**	GGCGGTGCCTATGTCTCA	AGGGTCTGGGCCATAGAA
**mIL-1β**	GCAACTGTTCCTGAACTCAACT	ATCTTTTGGGGTCCGTCAACT
**mCD68**	CAAGGGGGCTCTTGGGAACTA	GCTCTGATGTAGGTCCTGTTTG
**m18S**	TTGACTCAACACGGGAAACC	AGACAAATCGCTCCACCAAC

Supplementary Table 3: Particle size and zeta potential of liposomes.

Type	Particle size (nm)	Zeta Potential (mV)	Encapsulation efficiency
**RGD-Lip**	77.33±1.561	-17.5	/
**RGD-Lip/zileuton**	72.37±2.248	-14.8	70.42±0.722

Data are mean ± SD

Supplementary Figure 1

A

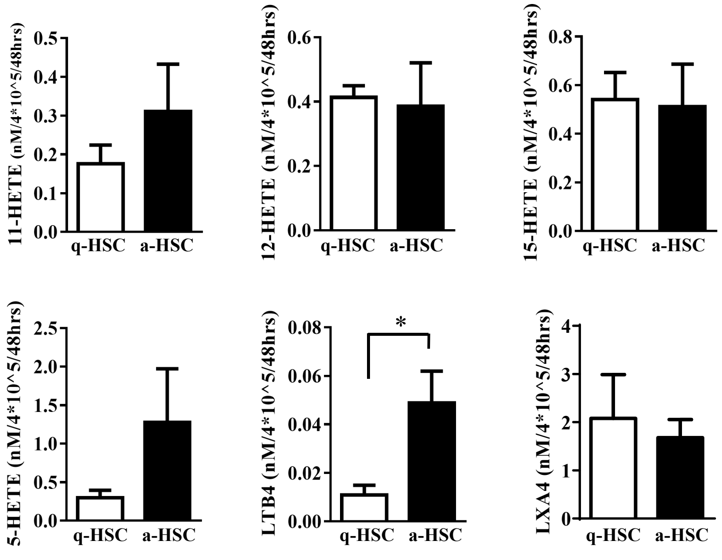

Supplementary Figure 2

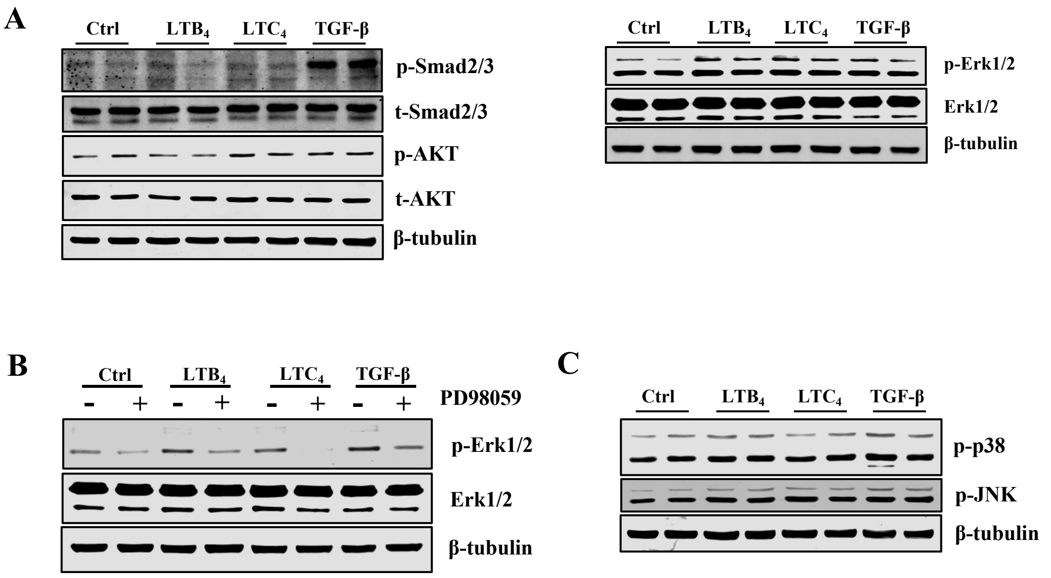

Supplementary Figure 3

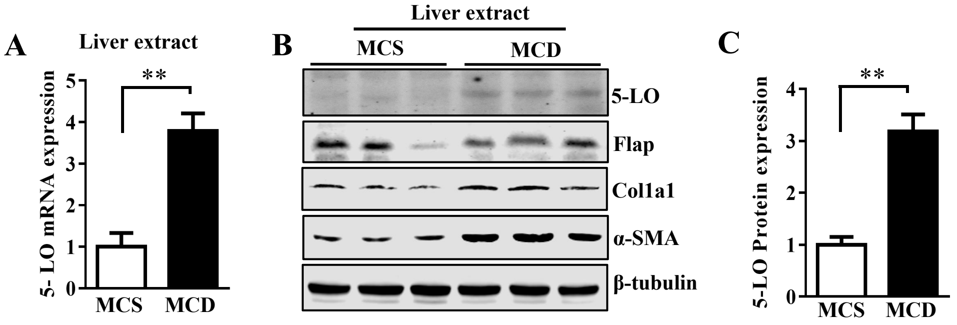

Supplementary Figure 4

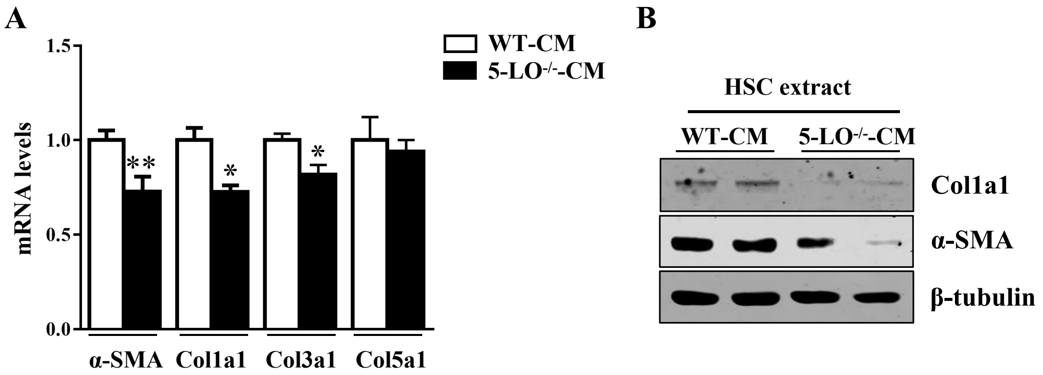

Supplementary Figure 5

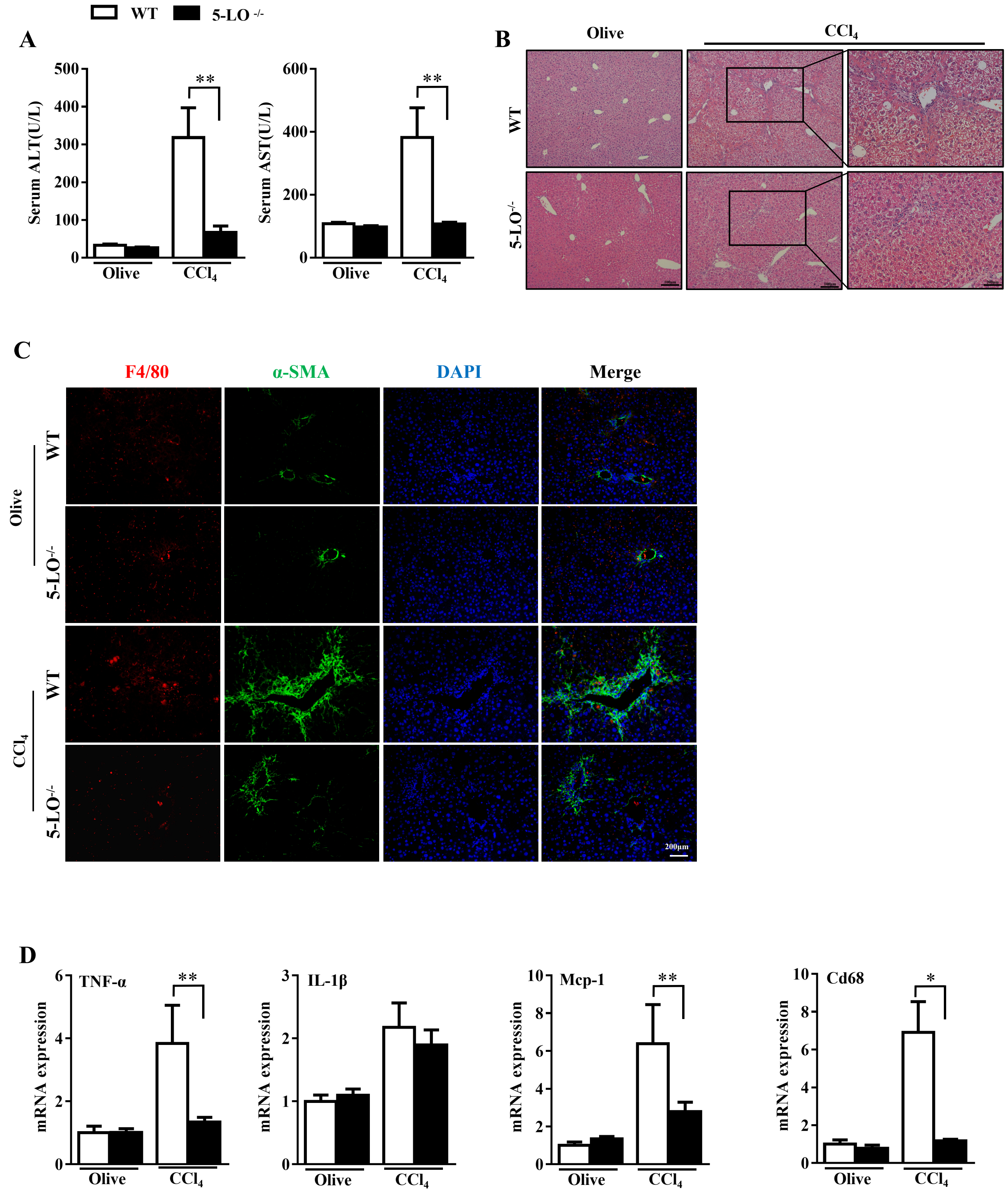

Supplementary Figure 6

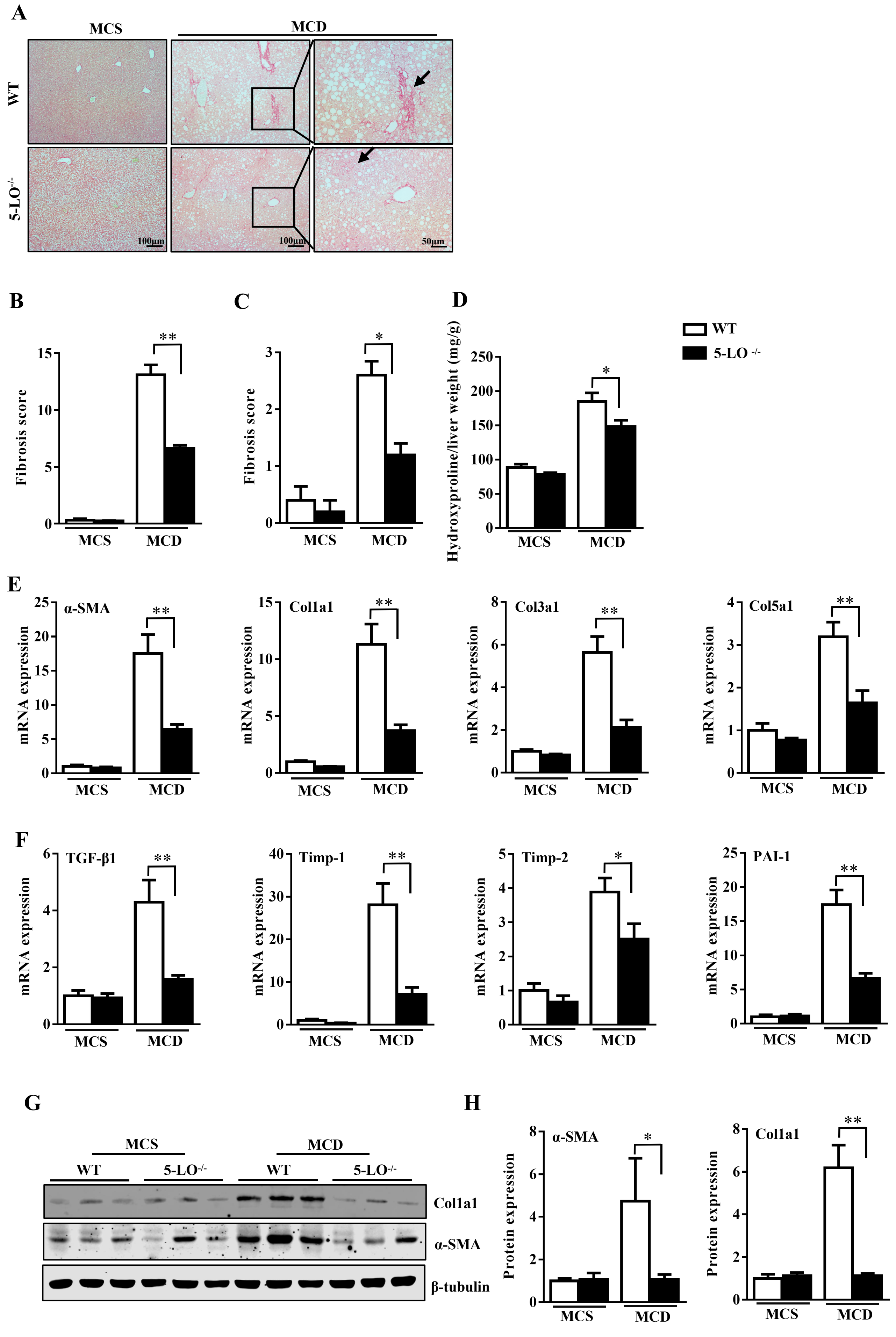

Supplementary Figure 7

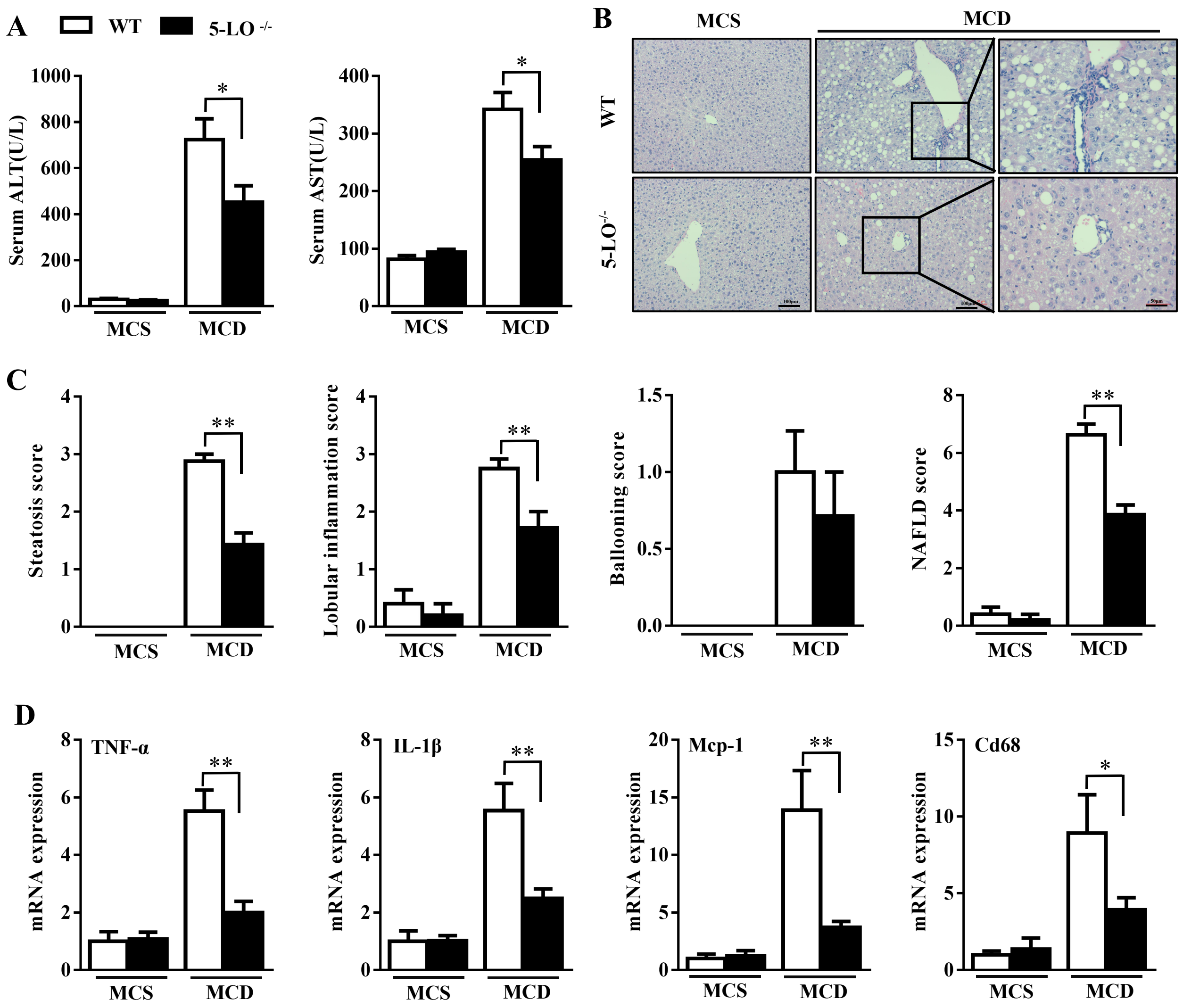

Supplementary Figure 8

A

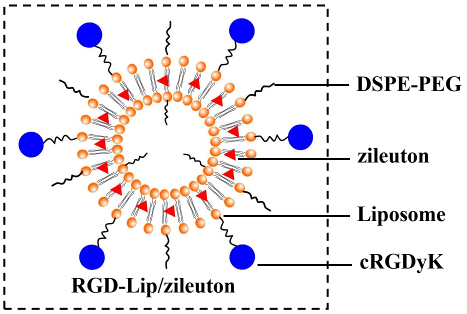

B

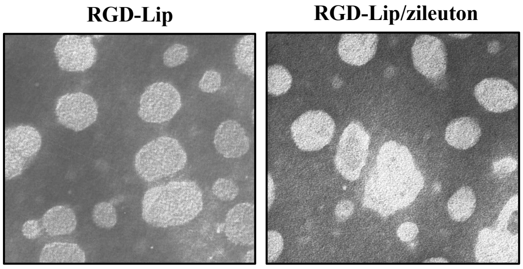

Supplementary Figure 9

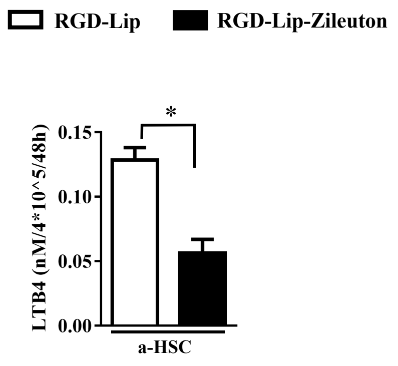

Supplementary Figure 10

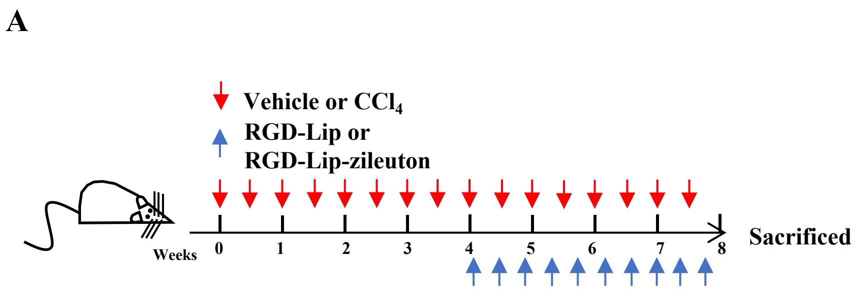

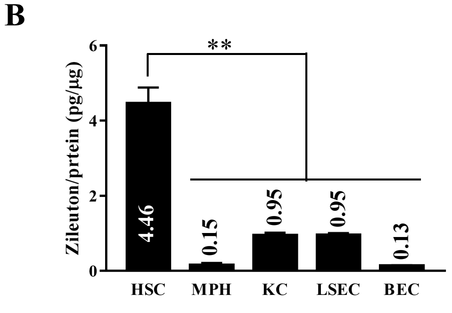

Supplementary Figure 11

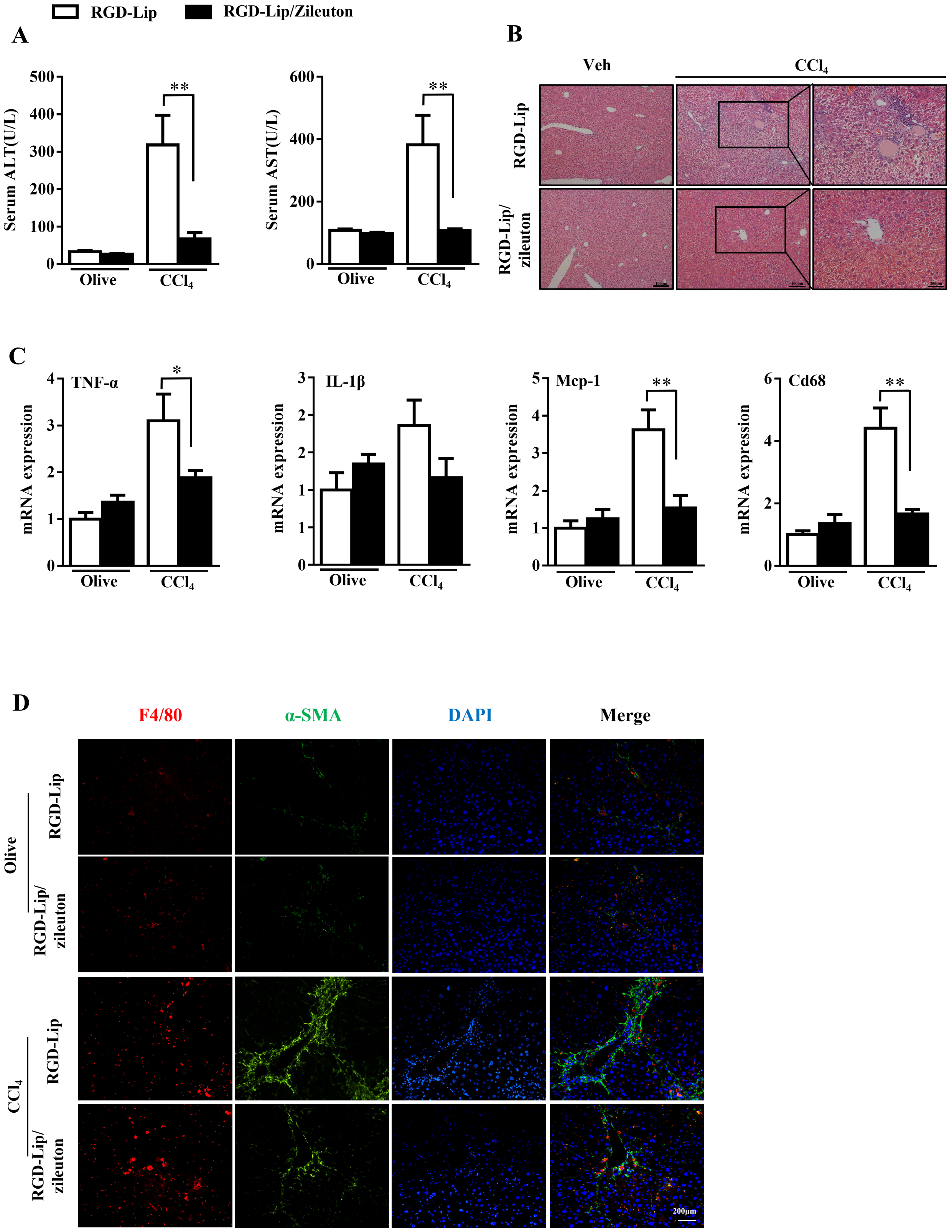

Supplementary Figure 12

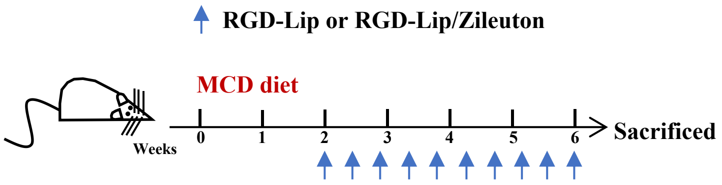

Supplementary Figure 13

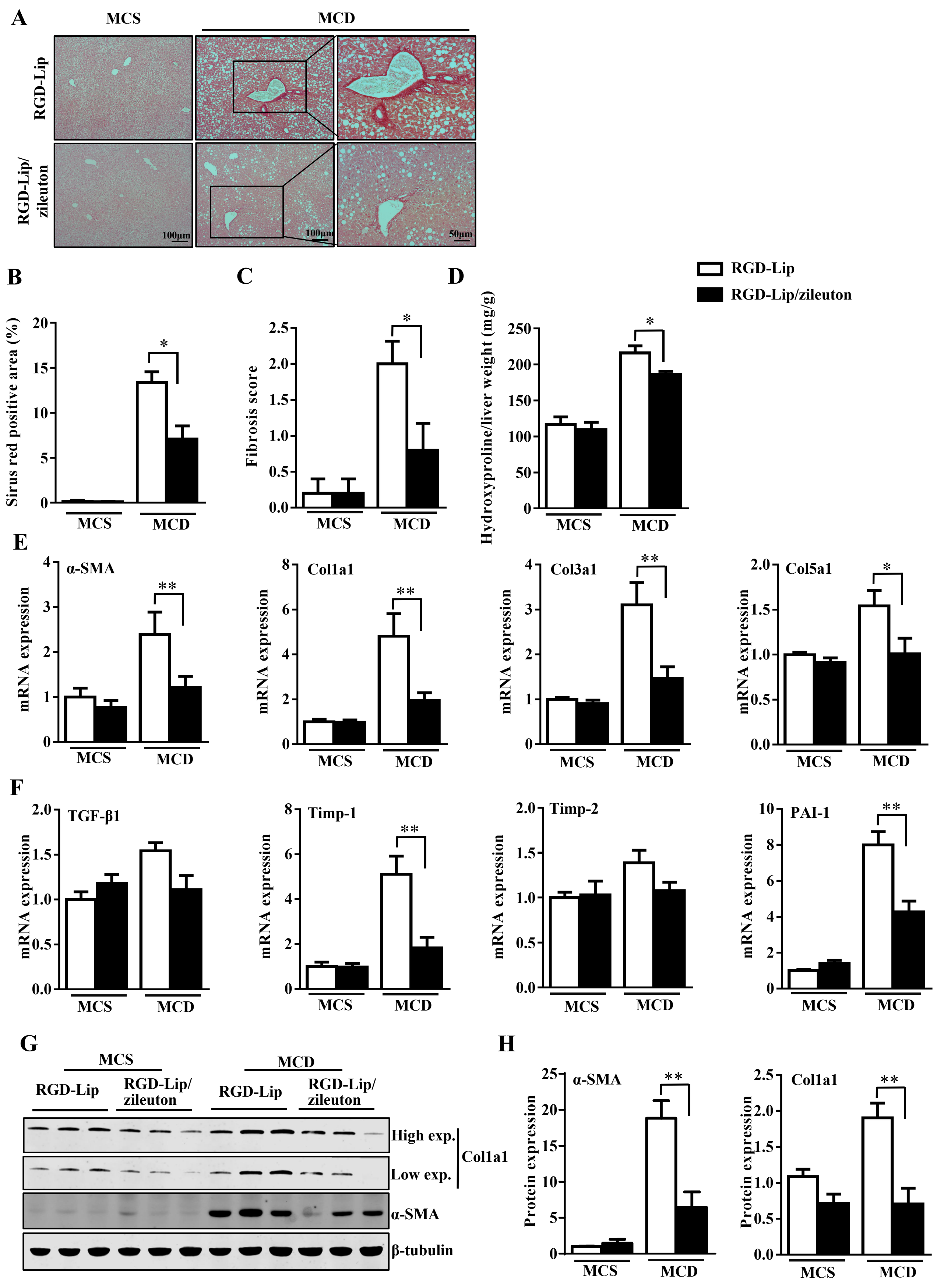

Supplementary Figure 14

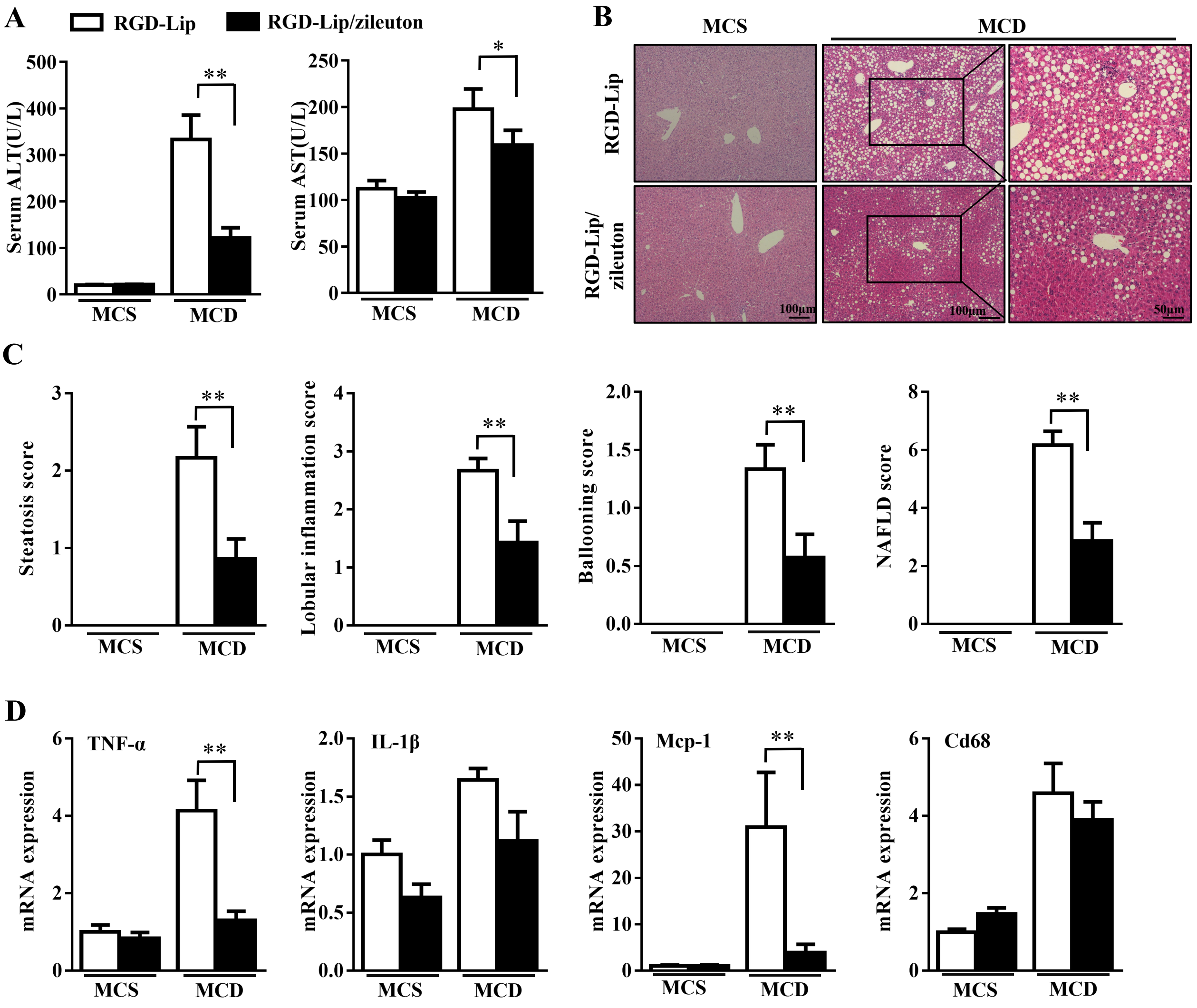

Supplementary Figure 15

A

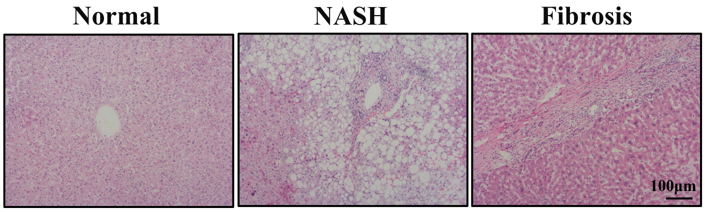

B

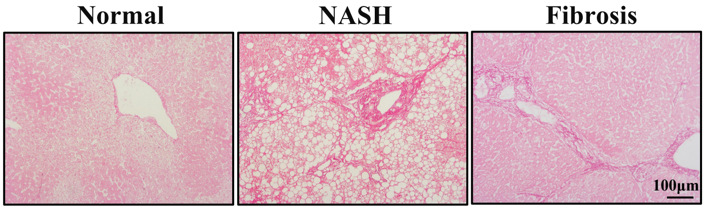

C

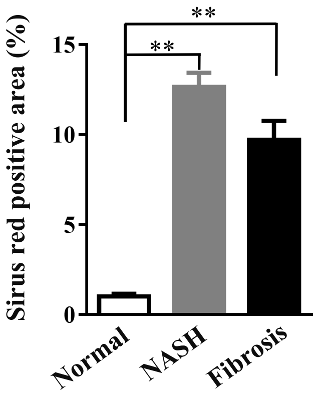

Supplement: Supplementary file 1 [file datasheet1.pdf]
